# Recent advances in the chemistry of ketyl radicals†

**DOI:** 10.1039/d0cs00358a

**Published:** 2021-03-23

**Authors:** Áron Péter, Soumitra Agasti, Oliver Knowles, Emma Pye, David J. Procter

**Affiliations:** Department of Chemistry, The University of Manchester Oxford Road Manchester UK david.j.procter@manchester.ac.uk

## Abstract

Ketyl radicals are valuable reactive intermediates for synthesis and are used extensively to construct complex, functionalized products from carbonyl substrates. Single electron transfer (SET) reduction of the C=O bond of aldehydes and ketones is the classical approach for the formation of ketyl radicals and metal reductants are the archetypal reagents employed. The past decade has, however, witnessed significant advances in the generation and harnessing of ketyl radicals. This tutorial review highlights recent, exciting developments in the chemistry of ketyl radicals by comparing the varied contemporary – for example, using photoredox catalysts – and more classical approaches for the generation and use of ketyl radicals. The review will focus on different strategies for ketyl radical generation, their creative use in new synthetic protocols, strategies for the control of enantioselectivity, and detailed mechanisms where appropriate.

## Abstract 2

Ketyl radicals are valuable reactive intermediates for synthesis. This review highlights exciting recent developments in the chemistry of ketyl radicals by comparing contemporary and more classical approaches for their generation and use.

## Graphical abstract

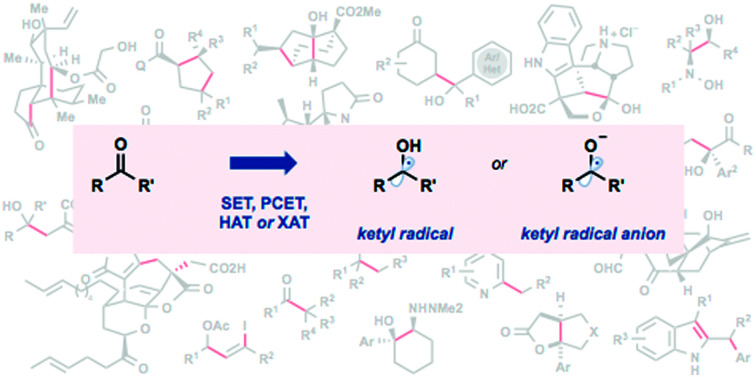

**Key learning points**

• The strategies available for ketyl radical generation.

• The structure and reactivity of ketyl radicals.

• How ketyl radicals can be generated under catalytic conditions.

• How the enantioselectivity of reactions involving ketyl radicals can be controlled.

• How ketyl radicals can be harnessed in target synthesis.

## 1. Introduction

**Fig. 1:**
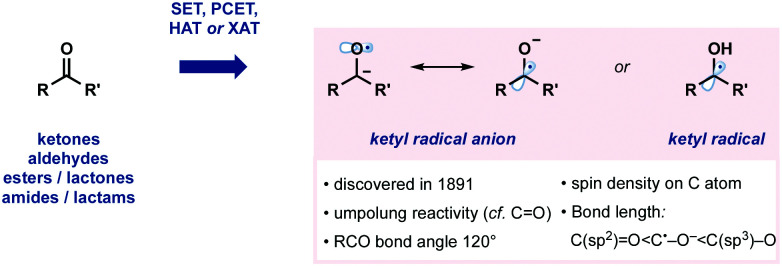
Some properties of ketyl radicals and their anions.

Ketyl radicals, and ketyl radical anions, are highly valuable, functionalized, reactive intermediates. Since their discovery in 1891, they have played a significant role in synthesis (Fig. 1).^1^ For example, ketyl radicals are readily generated from carbonyl compounds and thus play a pivotal role in strategies for carbonyl umpolung reactivity. Ketyl radicals and ketyl radical anions are one of the most well-studied reactive intermediates and are commonly exploited in powerful methods for C–C bond formation. A wide range of often complex molecular architectures is accessible by harnessing the generation and reaction of ketyl radicals in radical approaches that complement more usual ionic protocols. Recent years have seen the development of methodologies that generate and exploit ketyl radicals in innovative new ways. For example, single electron transfer (SET), proton-coupled electron transfer (PCET), hydrogen atom abstraction (HAT), and halogen atom abstraction (XAT) have all been used to access ketyl radicals (Fig. 1).

The structural properties of ketyl radicals, and their anions, closely resemble carbonyl compounds in terms of bond angle. In contrast, in ketyl radical anions, the C˙–O^−^ bond is longer than a C(sp^2^)=O, but shorter than a C(sp^3^)–O bond. DFT and other studies have shown that the spin density mainly resides on the carbon atom of ketyl radical anions.^1^

Covering the literature of the past 10 years, this tutorial review highlights exciting developments in the chemistry of ketyl radicals by comparing the varied contemporary – for example, using photoredox catalysts – and more classical approaches for the generation and use of ketyl radicals. The review will focus on different strategies for ketyl radical generation, their creative use in new synthetic protocols, strategies for the control of enantioselectivity, and the proposed mechanisms of reactions involving the reactive intermediates. The overview will be an invaluable introduction to the field and a useful resource for synthetic chemists wishing to rationally design new synthetic processes that exploit the unique chemistry of ketyl radicals.

## 2. Overview of ketyl radical generation

The aldehyde and ketone functional groups are fundamentally important handles for synthesis. In general, polarization of the carbon–oxygen double bond (C=O) results in an electrophilic centre at the carbon atom. Therefore, traditionally, these species were functionalised by strong nucleophiles, such as RMgX or RLi. The formation of ketyl radicals, or ketyl radical anions, possessing a *nucleophilic* carbon radical offers a means of inverting the reactivity of the starting carbonyl compound and using them in alternative C–C bond forming reactions with non-nucleophilic partners.

The formation of ketyl radicals by SET reduction does, however, suffer from a major limitation; the high reduction potential of aldehydes (*E*_p/2_ (benzaldehyde/benzaldehyde˙^−^) = −1.93 V *vs.* SCE in MeCN) and ketones (*E*_p/2_ (acetophenone/acetophenone˙^−^) = −2.11 V *vs.* SCE in MeCN). To overcome the high barriers to reduction, strong single electron reducing agents based on K,^2^ Zn,^3^ Mn,^4^ Ti,^5^ and Sn^6^ have been used. Arguably, samarium diiodide (SmI_2_), remains the reagent of choice for ketyl radical generation from aldehydes and ketones due to its selectivity, versatility, commercial availability, and ease of use.

With the advent of photoredox catalysis, new photochemical approaches for the generation of ketyl radicals from activated carbonyl compounds have emerged. Following excitation, SET from highly reducing photocatalysts to aldehydes and ketones forms ketyl radicals or ketyl radical anions. Attractively, ketyl radicals can be accessed without recourse to the use of radical initiators or stoichiometric metallic reductants. The resultant ketyl radicals can be used in various C–C bond forming reactions, including important ketyl–olefin couplings.

Homocoupling of ketyl radicals can lead to ‘pinacol’ by-products. However, this unwanted pathway can be suppressed by using a reactive, polarity-matched coupling partner, employing an excess of the coupling partner, or by maintaining a low concentration of ketyl radical anion. Intramolecular processes are less-likely to suffer from unwanted pinacol coupling.^7^

The utility of ketyl radicals is perhaps best illustrated by their privileged status in natural product total synthesis; this status arises from the high chemo-, regio-, and stereoselectivity of their reactions and the functionalized products that result. An ever-growing number of total syntheses of challenging targets have been unlocked by the chemistry of ketyl radicals. Furthermore, ketyl radicals have recently been exploited in enzymatic reactions^8^ and in polymerisations^9^ that deliver high value products.

## 3. Generation of ketyl radicals using metals, pseudo-metals and organocatalysts

Strong single-electron reductants, such as K,^2^ Zn,^3^ Mn,^4^ Ti^5^ and Sn,^6^ as well as electrochemical conditions, have been widely employed for the generation of ketyl radicals. The requirement for stoichiometric amounts of reductant can undermine the synthetic utility of these transformations. However, the use of SmI_2_ for ketyl radical formation has remained popular due to its versatility—a variety of carbonyl compounds can be converted to the corresponding ketyl radicals using the reagent – tuneability, commercial availability, and ability to control the stereochemical course of subsequent radical couplings by coordination to Lewis basic sites in substrates.^10–12^

**Scheme 1:**
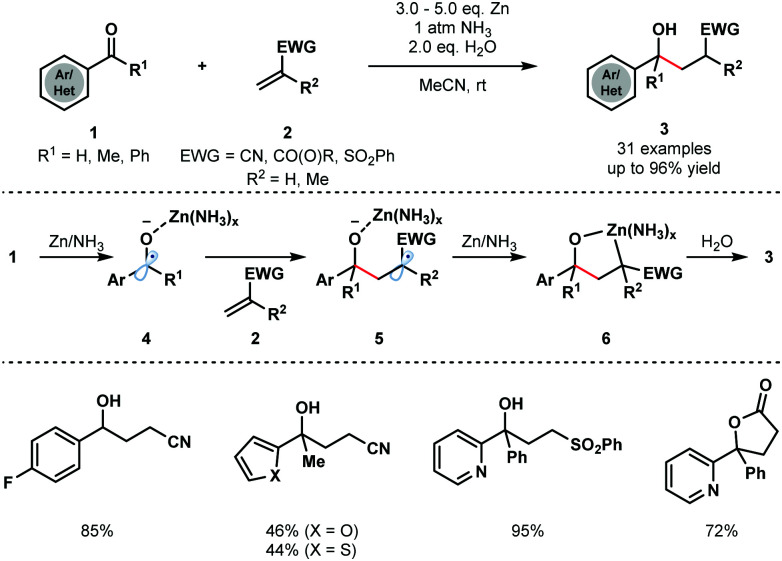
Zinc–NH_3_-mediated ketyl olefin couplings (Cheng, 2013).

In a classical approach, Cheng and co-workers reported the coupling of aryl/heteroaryl ketones and aldehydes **1** with a range of electron deficient alkenes **2**, including acrylates, acrylamides, acrylonitriles, and vinyl sulfones, to furnish γ-hydroxyl butyric acid derivatives **3** (Scheme 1).^13^ Importantly, the reaction does not require strong single-electron reducing agents, such as alkali metals, and instead uses inexpensive Zn metal. The process is compatible with a variety of substituted aryl and heteroaryl groups and provides straightforward access to simple γ-hydroxyl butyric acid derivatives. Interestingly, the Zn/H_2_O reducing system was used at room temperature under an atmosphere of NH_3_; Zn metal or a combination of Zn metal powder with other amines failed to deliver the desired coupling products.

Control experiments revealed that ammonia does not activate zinc but is necessary to form a more reducing Zn/NH_3_ complex. SET reduction of **1** furnishes zinc ketyl radicals **4** that add to the alkene substrates **2** to give radicals **5**. Further reduction is thought to then afford five-membered oxametallacycles **6** that undergo protonation to give the reductive coupling products **3**.

**Scheme 2:**
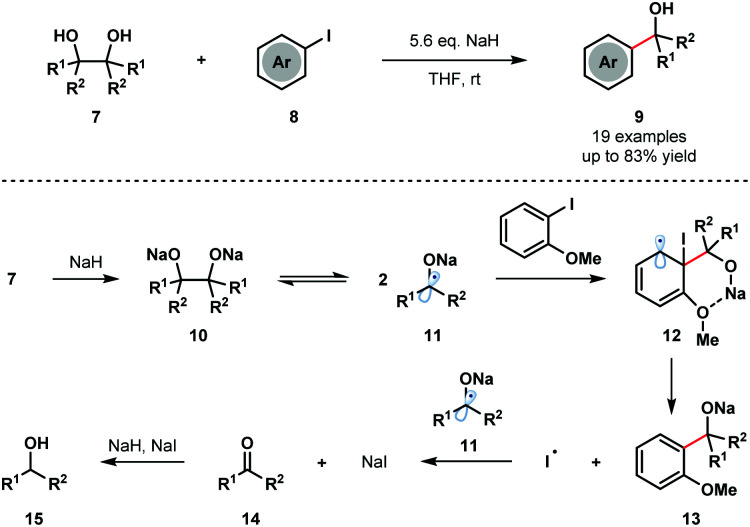
Cross-coupling of sodium ketyl radicals and aryl iodides to give tertiary benzylic alcohols (Studer, 2016).

Pinacol coupling typically involves reduction of a ketone or aldehyde precursor using a stoichiometric SET reductant, followed by homocoupling of the resultant ketyl radical anion. Reversibility of the pinacol coupling process has not previously been utilized in synthesis to generate ketyl radicals. For the first time, in 2016, Studer and co-workers exploited the reversibility of the pinacol coupling in a transition-metal-free C–C bond forming process involving aryl iodides **8** and ketyl radicals **11** (Scheme 2).^7^ The method was compatible with symmetrical and unsymmetrical pinacols and tolerated a range of aryl iodides bearing ortho heteroatom substituents. Upon deprotonation with NaH, readily accessible pinacols **7** were covered to Na-pinacolates **10** that are in equilibrium with sodium–ketyl radicals **11**. Addition of the ketyl radicals **11** to arenes **8**, facilitated by coordination of the ortho heteroatom substituents on the aryl iodide to the sodium cation, generates cyclohexadienyl radicals **12**. Fragmentation of **12** releases iodine radical and generates sodium alkoxides **13**, which upon protonation furnish the tertiary alcohol products **9** in good yield. The iodide radical is reduced by the ketyl radicals **11** to form ketones **14** and sodium iodide. The major byproducts, alcohols **15**, are thought to arise from the reduction of ketones **14** by the NaH/NaI couple.

**Scheme 3:**
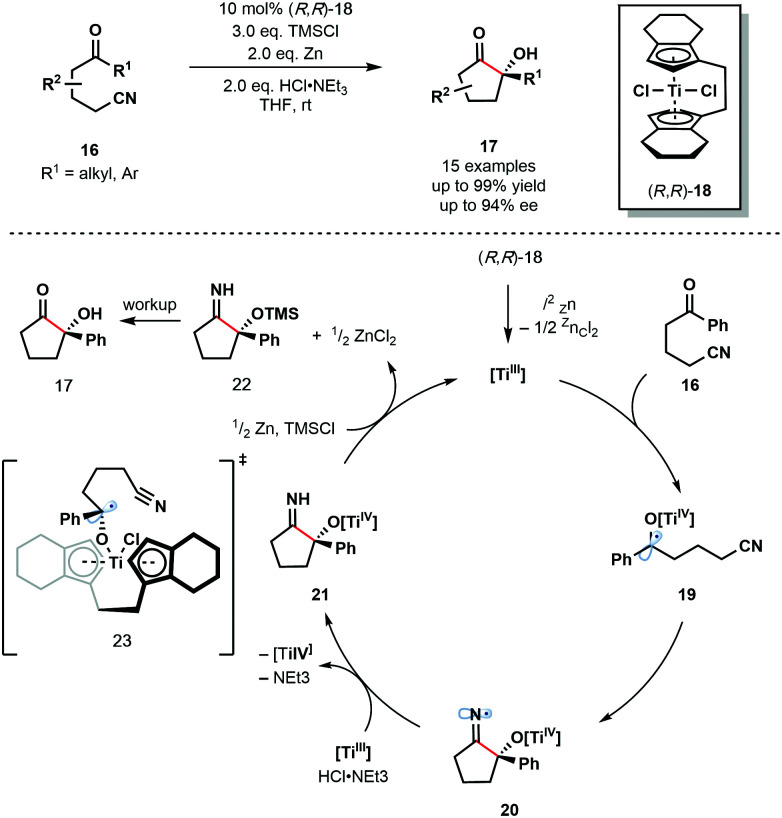
Ketyl radical intermediates in a Ti(iii)-catalyzed enantioselective cyclization of ketonitriles (Streuff, 2012).

In 2012, Streuff and co-workers reported the enantioselective reductive cyclization of ketonitriles **16** using the enantiopure Ti(iv)-precatalyst **18** (Scheme 3).^14^ Enantioselective Ti(iii)-mediated radical processes remain rare and the use of a catalytic amount of chiral transition metal catalyst alongside an inexpensive terminal reductant is attractive. The reaction was shown to tolerate aliphatic ketones, aryl ketones bearing electron-donating and electron-withdrawing substituents, and heteroaryl ketones.

*In situ* formation of the low-valent enantiopure Ti(iii) catalyst is followed by its coordination to the carbonyl group of substrates **16** and subsequent SET reduction to provide Ti(iv)–ketyl radicals **19**. Enantioselective radical 5-*exo*-dig cyclization involving the nitrile group and one enantiopic face of the ketyl radicals furnishes *N*-centred radicals **20**. These radicals are quickly reduced by the Ti(iii) catalyst, and the resultant anions protonated, to give the titanium(iv) alkoxide intermediates **21** which are subsequently quenched by TMSCl, liberating the α-oxygenated cyclic imines **22**. Finally, hydrolysis during workup furnishes ketone products **17**. The Ti(iii) catalyst is regenerated by the super-stoichiometric co-reductant, zinc dust. Transition state **23** was proposed to explain the origin of enantioselectivity in the reaction.

**Scheme 4:**
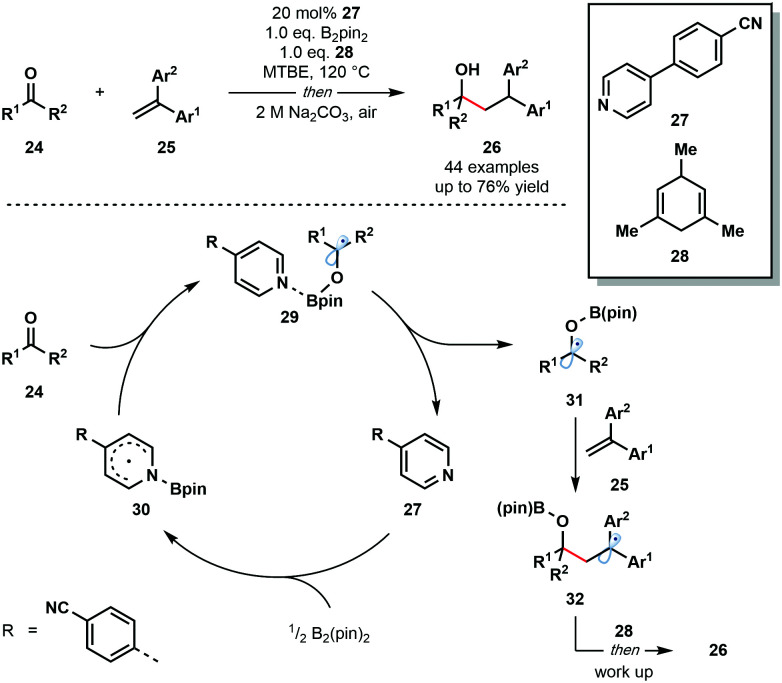
Carbonyl–alkene coupling featuring pyridine–boryl radicals (Li, 2018).

In 2018, Li and co-workers reported an intriguing route to ketyl radical derivatives that involves the SET reduction of ketones/aldehydes by a pyridine–boryl radical under thermal conditions (Scheme 4).^15^ This metal-free reductive coupling of aldehydes and ketones **24** with alkenes **25** boasts a broad substrate scope and good functional group compatibility. This method provides a new, metal-free approach for the generation of ketyl radical intermediates for exploitation in C–C bond formation.

The mechanism is thought to begin by the activation of the B–B bond of B_2_pin_2_ by a pyridine-derived organocatalyst **27** and formation of pyridine–boryl radical intermediate **30**. The addition of this radical to carbonyl substrates **24** generates pyridine complexed boron-containing ketyl radicals **29**. An alternative SET event between **30** and **24** was excluded based on DFT calculations. Dissociation of the pyridine catalyst allows ketyl radicals **31** to react with radical traps, such as **25**, to yield diaryl-stabilized radical intermediates **32**. Hydrogen atom abstraction from the sacrificial donor **28**, followed by work up, affords the final reductive coupling products **26**.

The lanthanide reductant samarium diiodide (SmI_2_) is a versatile and selective reagent for the generation of ketyl radical anions; coordination of Sm(iii) to the radicals often allows the stereochemistry of subsequent C–C bond-forming processes to be controlled as the metal coordinates to Lewis basic sites in the coupling partners. The use of water as an additive in reactions of SmI_2_ in THF allows the generation of previously inaccessible ketyl radicals, for example, those derived from lactones and other aliphatic carboxylic acid derivatives. This is due to the increased reduction potential of the SmI_2_–H_2_O system (*E*^red^_1/2_ (Sm(iii)/Sm(ii)) = −1.3 V *vs.* SCE in THF/DME), compared to SmI_2_ alone (*E*^red^_1/2_ (Sm(iii)/Sm(ii)) = −0.89 V *vs.* SCE in THF), and the H_2_O coordinated to Sm providing a proximal proton source for the quenching of reactive intermediates. It has also been proposed that PCET is involved in reactions of SmI_2_–H_2_O. Recent studies have shown that this reagent system can also be used to generate unusual ketyl radical anions by SET reduction of the carbonyl groups in esters and amides.^10–12^ Inorganic additives (*e.g.* LiCl, LiBr, NiCl_2_, and FeCl_3_) and organic additives (*e.g.* HMPA, TPPA, and other Lewis basic ligands) are commonly used in conjunction with proton sources (MeOH, *t*-BuOH, H_2_O) to fine-tune the selectivity and reactivity of SmI_2_. Although the reagent has been used in catalytic amounts in a handful of studies, the requirement for a super-stoichiometric terminal reductant has limited widespread uptake of these catalytic methods.^16^ Against this backdrop, Procter and co-workers have recently reported efficient SmI_2_-catalyzed cyclizations that involve the generation of ketyl radical anions by SET reduction of ketone carbonyl groups by SmI_2_ and that proceed under mild conditions and in the absence of stoichiometric co-reductants.^17^

**Scheme 5:**
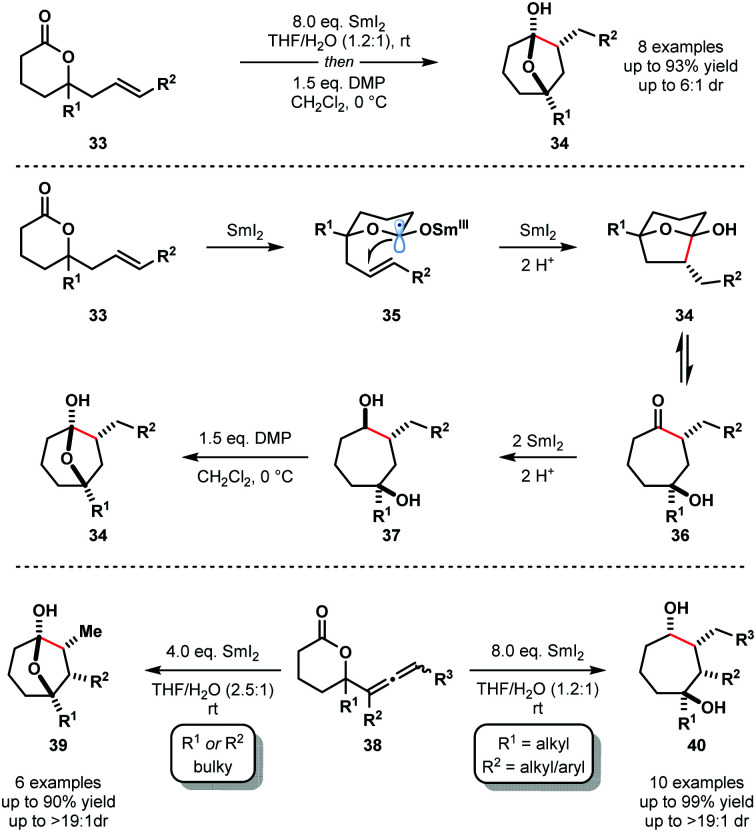
Ketyl radicals in lactone–alkene couplings using SmI_2_–H_2_O (Procter, 2012).

In 2012, Procter and co-workers reported the reductive cyclization of alkenyl-, alkynyl-, and allenyllactones to form oxygenated cycloheptanes using the SmI_2_–H_2_O system; allenyllactone substrates selectively produced cycloheptanes bearing three new stereocentres (Scheme 5).^18^ A variety of substituents on the lactone ring, and on the allene, were tolerated in these processes and products were obtained in good yield and with excellent diastereocontrol. The proposed mechanism for the reductive cyclization of alkenyllactones and allenyllactones proceeds *via* coordination of SmI_2_ to **33**, followed by SET reduction of the lactone carbonyl, generating Sm(iii) ketyl radicals **35**. 5-*Exo*-trig cyclization and subsequent reduction by another equivalent of SmI_2_ affords hemiketal intermediates **34**, which are in equilibrium with their ring-opened forms **36**. Further SET reduction of these intermediates gives diols **37** upon protonation. The reductive cyclization of allenyllactones **38** is postulated to proceed in a similar fashion, to give, in this case, cycloheptanone intermediates that undergo conjugate reduction to give hemiketals **39** or complex cycloheptan-1,4-diols **40**.^18^

**Scheme 6:**
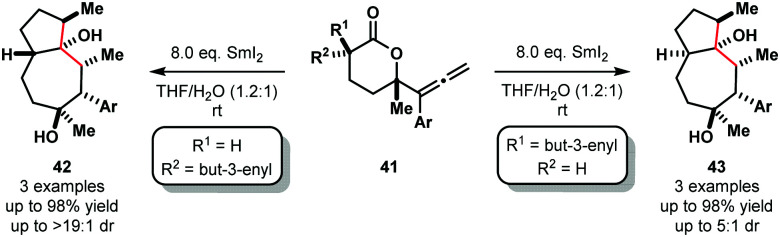
Ketyl radicals in lactone cascade cyclizations to give fused bicycles using SmI_2_–H_2_O (Procter, 2012).

Furthermore, selective cascade processes of alkenyl and allenyllactones can be used to generate complex bicyclic architectures **42** and **43** (Scheme 6). In these lactone cyclization cascades, the second Sm(iii) ketyl radical intermediates encountered along the mechanistic pathway – *i.e.* those generated from ketone intermediates like **36** – are intercepted by tethered alkenes, furnishing bicyclic tertiary alcohols **42** or **43** upon work up.^18^

**Scheme 7:**
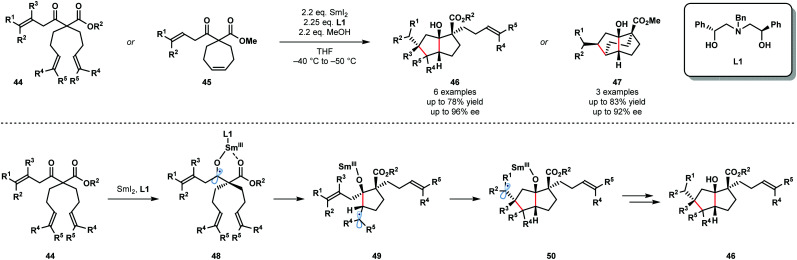
Enantioselective cyclization cascades of Sm(iii) ketyl radicals (Procter, 2017).

The synthetic potential of enantioselective radical cyclization cascades remains largely unexplored. In particular, the use of chiral ligands to exert enantiocontrol in SmI_2_-mediated reactions, including cascade reactions, is a long-standing challenge. In 2017, Procter and co-workers reported the SmI_2_-mediated, enantioselective desymmetrizing ketyl–olefin radical cyclizations and cyclization cascades of dienyl β-ketoesters (Scheme 7).^19^ Readily available, recyclable, enantiopure aminodiol ligand **L1** facilitated the enantioselective cyclization of substrates **44** and **45** and the efficient formation of **46** and **47**. This is the first example of a highly enantioselective SmI_2_-mediated ketyl–olefin cyclization. MeOH was used as a sacrificial proton source to preserve the integrity of the chiral aminodiol ligand **L1**. Furthermore, MeOH was found to increase the level of enantiocontrol observed and likely coordinates to the samarium metal centre. Substrates containing up to five new stereocentres were isolated in good yield and with high enantiopurity. The proposed mechanism begins by coordination of the two-point-binding substrates, **44** or **45**, to SmI_2_ bearing chiral ligand **L1**. Next, SET from Sm(ii) to the ketone moiety affords Sm(iii) ketyl radicals **48**, which undergo enantiodetermining cyclization to give radicals **49**. Trapping of radical intermediates **49** by the proximal tethered alkene yields intermediates **50** that undergo further SET reduction and protonation to give **46**.

**Scheme 8:**
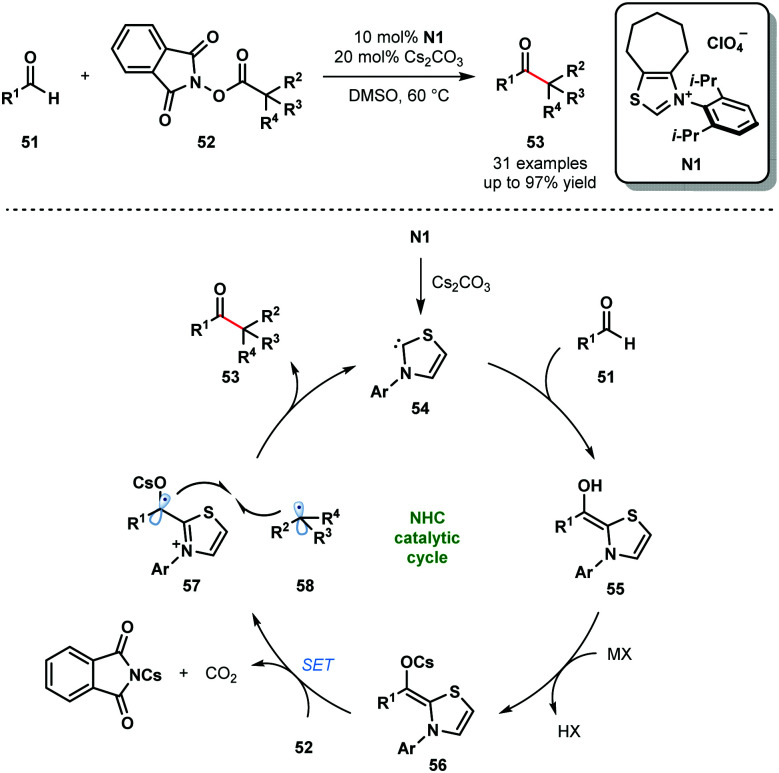
Ketyl radicals in NHC-catalysed decarboxylative coupling of activated carboxylic acids and aldehydes (Ohmiya, 2019).

N-Heterocyclic carbenes (NHCs) are also known to facilitate radical reactions that involve ketyl radicals. In particular, deprotonated Breslow intermediates, generated from aldehydes using NHCs, are useful SET reductants. In 2019, the Ohmiya group reported a decarboxylative coupling of aryl aldehydes **51** and redox-active esters **52** to produce sterically congested aryl alkyl ketones **53** (Scheme 8).^20^ This methodology allows for the use of widely-available carboxylic acids as coupling partners in radical–radical couplings involving ketyl radicals. The selectivity for products of cross-coupling, rather than homo-coupling, is striking. The process tolerates a wide range of functional groups in both partners, however, only tertiary and secondary carboxylic acid partners were used and the scope with aliphatic aldehydes was not explored. The reaction is thought to begin by deprotonation of **N1** to yield the active catalytic NHC species **54**. The neutral Breslow intermediates **55** are formed by addition of **54** to aldehydes **51**. Deprotonation then yields the reducing intermediates **56** (*E*^red^_1/2_ (**57**/**56**) = −0.96 V *vs.* SCE in MeCN) and SET reduction of **52** provides alkyl radicals **58** and ketyl radicals **57**. Redox-active esters **52** (*E*^red^_1/2_ (**52**/**52**˙^−^) ≤ −1.28 V *vs.* SCE in MeCN) are likely activated by Cs^+^ prior to SET reduction. Finally, radical–radical coupling between **57** and **58**, followed by the elimination of **54**, affords congested ketones **53** and closes the catalytic cycle.

## 4. Light induced ketyl radical generation

Visible light-mediated photoredox catalysis is now established as a powerful tool to access ketyl radicals,^21–26^ thus complementing traditional approaches to the reactive intermediates. Upon visible light excitation, photocatalysts can engage in SET processes that generate radicals, thus avoiding the use of classical radical initiators or stoichiometric metal reagents.

In 1978, Kellogg *et al.* reported the use of Ru^II^(bpy)_3_Cl_2_ as a catalyst for the SET reduction and fragmentation of aryl carbonyl compounds bearing α-sulfonium groups.^27^ The process likely proceeds through the generation of ketyl radicals. Since then, visible light-mediated photoredox catalysis has become widely recognized as an efficient strategy for the production of ketyl radicals from aldehydes and ketones under mild conditions. The resulting ketyl radicals have been exploited in a diverse array of C–C bond forming reactions that deliver high-value, and often complex, molecular architectures. This section of the tutorial review highlights recent, significant advancements made in the chemistry of photocatalytically generated ketyl radicals.

Most mechanisms in this section are depicted as closed photocatalytic cycles, as suggested by the original authors. However, it is important to note that, in the absence of quantum yield measurements, for example, radical chain processes can not be ruled out.^28^

### 4.1 Intramolecular transformations

**Scheme 9:**
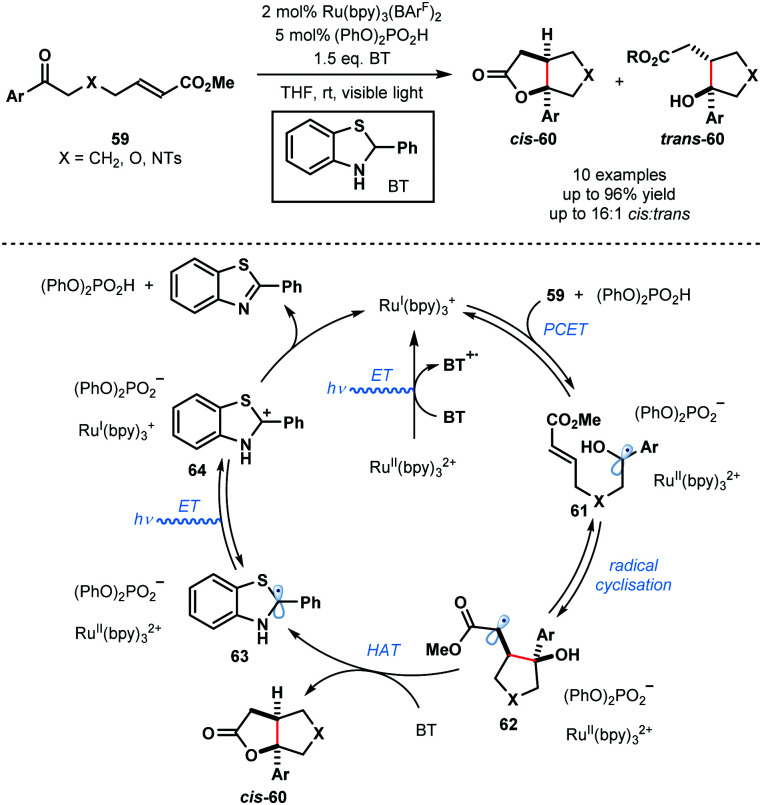
Proton-coupled electron transfer in photocatalytic ketyl–olefin cyclisations (Knowles, 2013).

In 2013, Knowles and co-workers reported a catalytic intramolecular ketyl–olefin coupling reaction that is facilitated by proton coupled electron transfer (PCET) (Scheme 9).^29^ This seminal study constitutes the first synthetically useful example of ketyl radical generation using visible-light. The cyclisation was achieved using catalytic Ru(bpy)_3_(BArF)_2_ and diphenyl phosphoric acid, and the terminal reductant, 2-phenyl-dihydrobenzothiazoline (BT), in the presence of visible light irradiation, and was used to deliver substituted cyclopentanes, tetrahydrofurans and pyrrolidines. The scope focuses on aryl alkyl ketones and is limited to activated alkenes. Visible light-mediated photoexcitation of the ground state photocatalyst, Ru^II^(bpy)_3_^2+^, followed by reductive quenching of the excited photocatalyst, Ru^II^(bpy)_3_^2+^*, by BT forms the SET reductant Ru^I^(bpy)_3_^+^. Concurrently, the Brønsted acid co-catalyst activates the ketone functionality in **59** by a hydrogen bonding interaction. Subsequent PCET delivers ketyl radical intermediates **61**. The resultant ketyl radicals **61** undergo facile intramolecular radical addition onto the double bond forming a stabilised radical species **62**. Hydrogen atom transfer from BT to **62** delivers products **60** and radical **63**. Ru^II^(bpy)_3_^2+^ is reduced by radical **63** to regenerate Ru^I^(bpy)_3_^+^ and carbocationic intermediate **64**. Finally, deprotonation of **64** by the phosphate anion regenerates the Brønsted acid co-catalyst and closes the catalytic cycle.

**Scheme 10:**
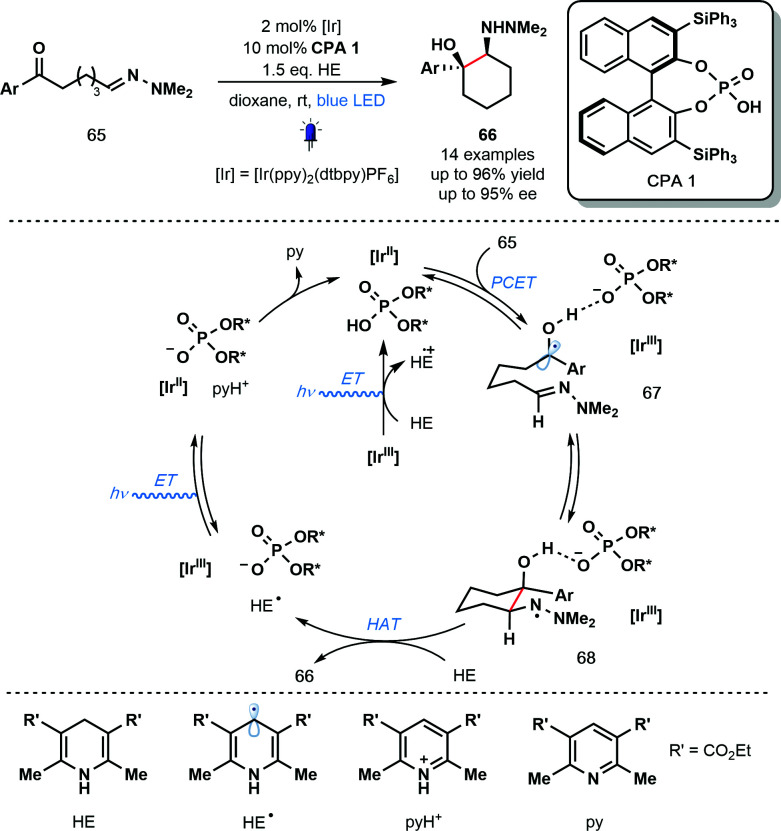
Photocatalytic enantioselective aza-pinacol cyclization employing a chiral phosphoric acid and exploiting PCET (Knowles, 2013).

In the same year, Knowles and co-workers reported the use of PCET for ketyl radical generation in enantioselective catalysis (Scheme 10).^30^ The intramolecular reductive coupling of ketones and hydrazones, involving ketyl radicals **67**, used a chiral phosphoric acid (CPA) catalyst to control the enantioselectivity of the reaction. A range of *syn*-1,2-hydrazo alcohols is accessible with excellent levels of diastereo- and enantiocontrol using catalyst CPA 1 in combination with the photoredox catalyst Ir^III^(ppy)_2_(dtbpy)PF_6_. This is the first example of an enantioselective, photoredox aza-pinacol coupling. Building on their earlier work (Scheme 9), the reaction is thought to be initiated by the off-cycle excitation of the photocatalyst to give [Ir^III^]* which is then converted to [Ir^II^] by the sacrificial reductant, HE (Hantzsch ester). Concerted PCET from [Ir^II^] to the complex of ketones **65** and **CPA 1** leads to the formation of phosphate bound ketyl radical intermediates **67**. Enantioselective radical cyclization, followed by HAT from HE affords the enantioenriched, cyclic products **66** and HE˙. Excitation of [Ir^III^] and oxidation of HE˙ by the resulting [Ir^III^]* produces pyH^+^ and regenerates the catalytically active [Ir^II^]. Finally, the conjugate anion of CPA 1 deprotonates pyH^+^ and returns the CPA 1 catalyst.

**Scheme 11:**
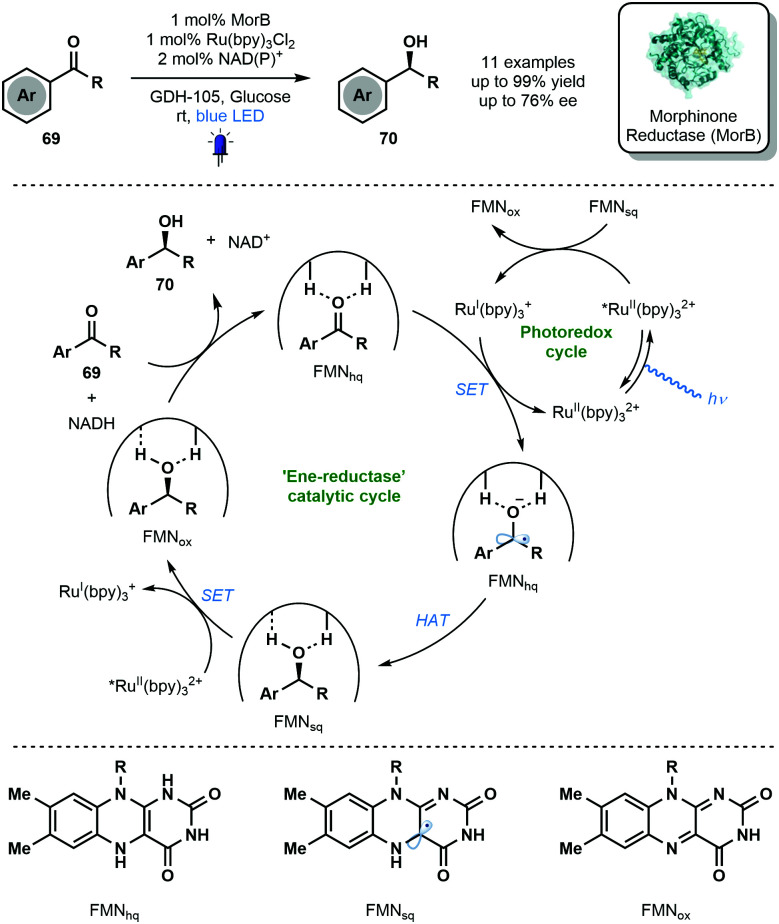
Enantioselective reduction of aromatic ketones through the integration of enzymatic and photoredox catalysis (Hyster, 2019).

In 2019, Hyster and co-workers reported the use of the flavin-dependent ene-reductase, MorB (Morphinone Reductase), in conjunction with Ru(bpy)_3_Cl_2_ and visible light to achieve the enantioselective reduction of aryl ketones *via* enzyme-bound ketyl radical anion intermediates (Scheme 11).^8^ Mechanistic studies indicated that binding of the carbonyl group by the active site of MorB lowers the reduction potential of the ketone moiety and thus facilitates its reduction by Ru^I^(bpy)_3_^+^. HAT from FMN_hq_ then quenches the resulting ketyl radical anion. The photocatalytic cycle is closed by an electron transfer process between FMN_sq_ and Ru^II^(bpy)_3_^2+^*. The novel marriage of photo- and bio-catalysis afforded enantioenriched alcohols **70** in good to excellent yield with moderate enantioselectivity. Despite modest scope and selectivity, this exciting approach paves the way for future marriages of photo- and bio-catalysis featuring ketyl radicals.

**Scheme 12:**
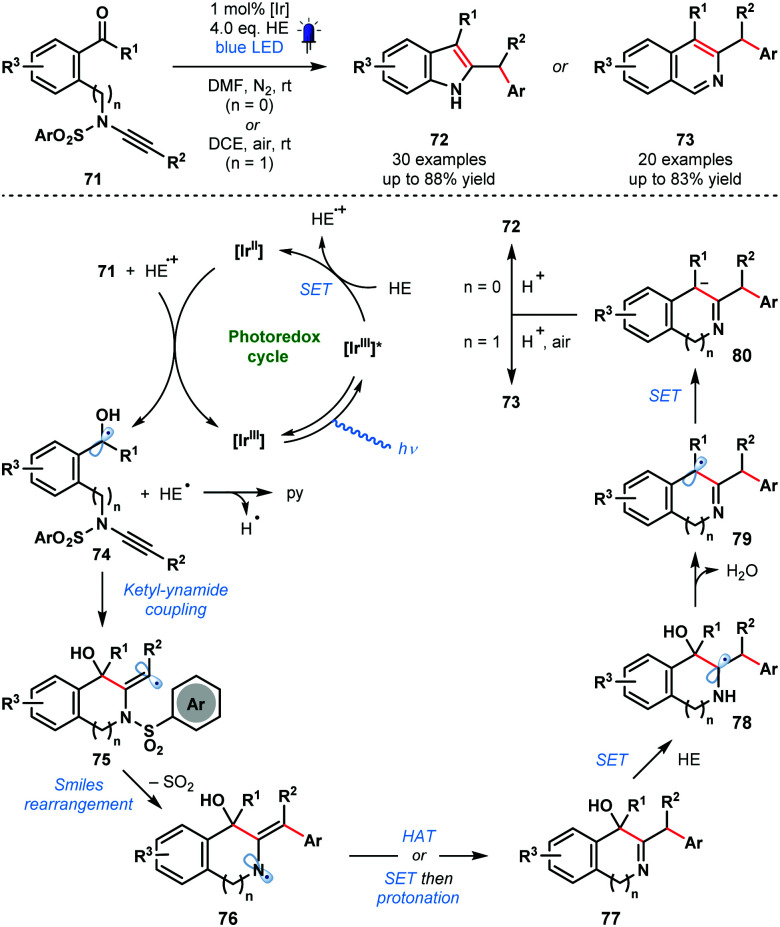
Photocatalytic synthesis of substituted indoles and isoquinolines by ketyl–ynamide coupling and radical Smiles rearrangement (Ye, 2020).

In 2020, Ye and co-workers reported a photocatalytic ketyl–ynamide coupling that triggers a radical Smiles rearrangement and allows efficient access to a range of 2,3-substituted indoles (**72**) and 3,4-substituted isoquinolines (**73**) (Scheme 12).^31^ This is the first radical Smiles rearrangement featuring ynamides. The process tolerates a range of important substituents, including, halide, cyano, methoxy, and trifluoromethyl. The postulated mechanism begins with reduction of **71** by [Ir^II^] and formation of ketyl radicals **74**. Regioselective ketyl–ynamide coupling generates alkenyl radicals **75** and triggers a Smiles rearrangement to give **76**. Subsequent steps, featuring a number of SET events, yield products **72** or **73***via* intermediates **77–80**.

### 4.2 Intermolecular couplings

**Scheme 13:**
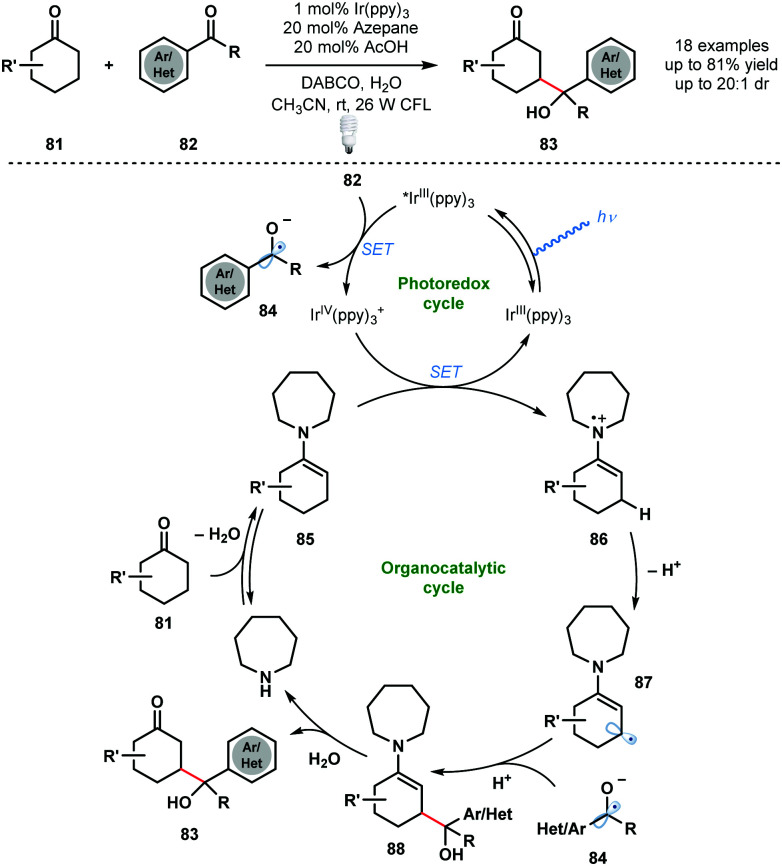
Dual photocatalytic and organocatalytic, direct β-functionalization of cyclic ketones (MacMillan, 2013).

An early dual photocatalytic and organocatalytic process was developed by MacMillan and co-workers for the direct β-functionalisation of cyclic ketones using aryl ketones (Scheme 13).^32^ A range of substituted aryl ketones **82** served as coupling partners with cyclic ketones **81**, in the presence of 1 mol% Ir^III^(ppy)_3_ and 20 mol% azepane, to afford γ-hydroxyketones **83** in high yield. Importantly, this method complements traditional ketone functionalisation at the carbonyl carbon and at the α-position. Furthermore, selective radical–radical cross-coupling is observed and issues of homo-coupling are avoided. Oxidative quenching of the excited photocatalyst Ir^III^(ppy)_3_* by the acid-activated aryl ketones **82** generates ketyl radical anions **84** and Ir^IV^(ppy)_3_^+^. Enamines **85**, formed by condensation of azepane with ketones **81**, reduce Ir^IV^(ppy)_3_^+^ and close the photocatalytic cycle. Deprotonation of **86** generates **87** and radical–radical coupling between ketyl radical anion **84** and **87** forms intermediate **88**. Subsequent hydrolysis of **88** liberates the β-functionalised cyclic ketone products **83** and regenerates azepane.

**Scheme 14:**
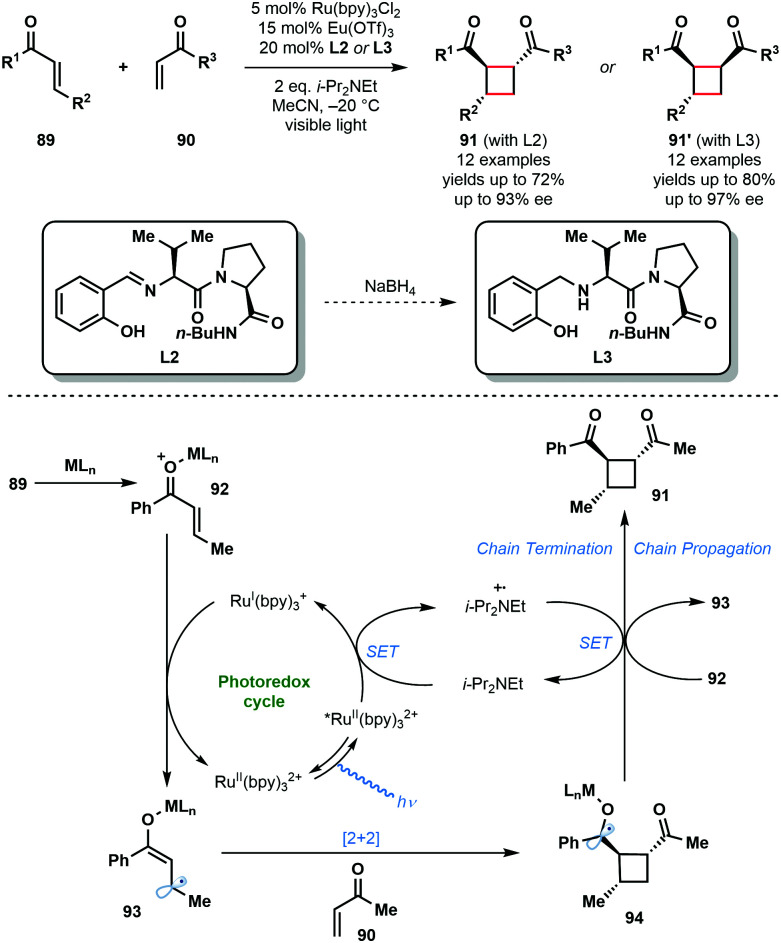
Enantioselective [2+2] photocycloaddition involving α,β-unsaturated ketones and using a transition–metal photocatalyst and Lewis acid co-catalyst. (Yoon, 2014).

In 2014, Yoon and co-workers reported the intermolecular asymmetric [2+2] photocycloaddition of α,β-unsaturated ketones, **89** and **90**, exploiting a dual catalyst system involving a photocatalyst and a chiral Lewis acid catalyst (Scheme 14).^33^ The major challenge involved in the development of an enantioselective [2+2] photocycloaddition reaction is the facile uncatalyzed, racemic background reaction mediated by light. By employing a photocatalyst, Ru^II^(bpy)_3_Cl_2_, that adsorbs at wavelengths where the substrates are not activated, and by using a Lewis acid to activate the enones towards SET and stabilize the resulting radical species, the racemic background reaction was overcome. Thus, using a Lewis acid, such as Eu(OTf)_3_, bearing a chiral ligand, **L2** or **L3**, promoted the enantioselective [2+2] reaction. The reaction mechanism starts with the photoexcitation of Ru^II^(bpy)_3_^2+^ to Ru^II^(bpy)_3_^2+^*. Reductive quenching of the excited state photocatalyst by i-Pr_2_NEt affords i-Pr_2_Net˙^+^ and Ru^I^(bpy)_3_^+^. Ru^I^(bpy)_3_^+^ then reduces the Lewis acid activated enone (**92**) by SET and produces delocalised ketyl radical anion (**93**). Subsequently, enone radical anion **93** undergoes enantioselective [2+2] cycloaddition with **90** to produce ketyl radical intermediate **94**. Ketyl radical **94** is converted to products **91** or **91′** either by reduction of i-Pr_2_Net˙^+^ or by reduction of another molecule of **92** in a chain propagation step.^28^

**Scheme 15:**
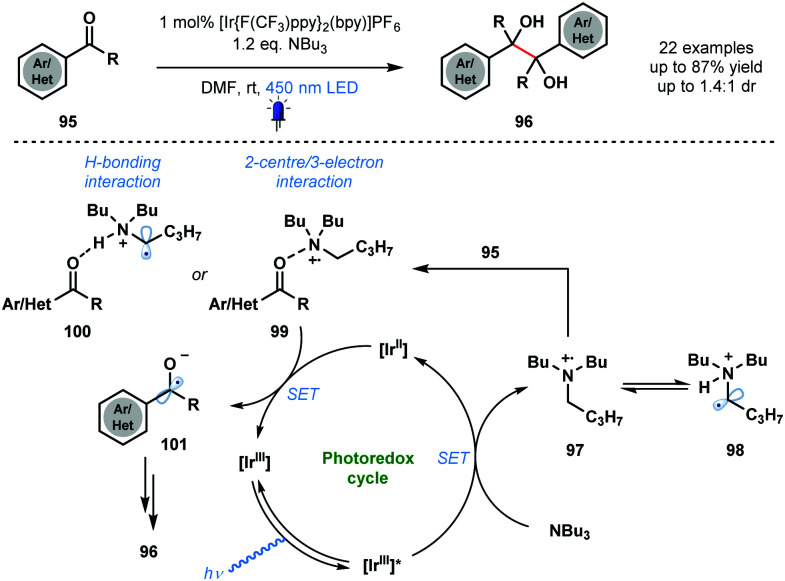
Photocatalytic homo-coupling of aldehydes and ketones (Rueping, 2015).

In 2015, Rueping and co-workers utilised photocatalytically generated ketyl radical anions, in conjunction with carbonyl activation by an amine radical cation, in the homo-coupling of aldehydes and ketones (Scheme 15).^34^ The mild method provides simple and convenient catalytic access to symmetrical pinacols **96** and diamines with good functional group tolerance. A variety of vicinal diols was synthesized in moderate to excellent yield using the approach. The proposed mechanism begins with quenching of the excited photocatalyst [Ir^III^]* by NBu_3_, the sacrificial reductant. Amine radical cation **97** is then thought to activate the weakly basic carbonyl groups of ketones **95** through formation of complexes **99** or **100**. Ketyl radical anion **101** is formed after SET reduction by [Ir^II^] and dimerization followed by protonation affords the vicinal diol products **96**. The proposal of a dual role for NBu_3_ in the transformation – sacrificial electron donor and substrate activator – is noteworthy.

**Scheme 16:**
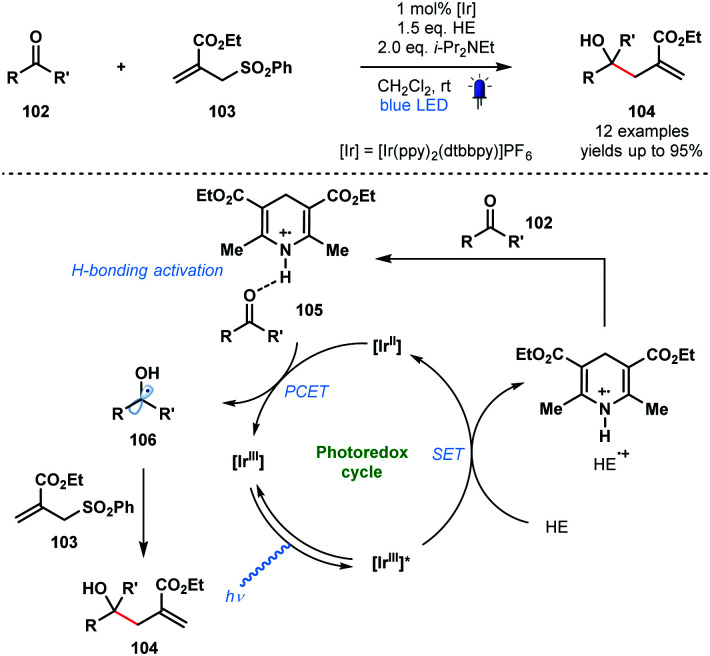
Photocatalytic allylation of aldehydes and ketones involving ketyl radicals (Chen, 2016).

In 2016, in a related process, Chen and co-workers reported a visible light-mediated ‘polarity-reversed’ allylation reaction of aldehydes and ketones that involves ketyl radicals (Scheme 16).^35^ These are the first visible-light induced, general allylation reactions of ketyl radicals. A Hantzsch ester is thought to act as both an electron/proton donor and as the activator of the carbonyl group in this transformation. After reductive quenching of the excited photocatalyst, HE˙^+^ activates the aldehyde/ketone partner **102** by a hydrogen bonding interaction prior to PCET and formation of the ketyl radical intermediates **106**. Intermolecular radical addition of **106** to allyl sulfones **103**, and radical elimination of the phenylsulfonyl group, yields adducts **104**. A quantum yield (*ϕ*) of 0.8 was calculated for the reaction and a closed catalytic cycle was therefore proposed.

**Scheme 17:**
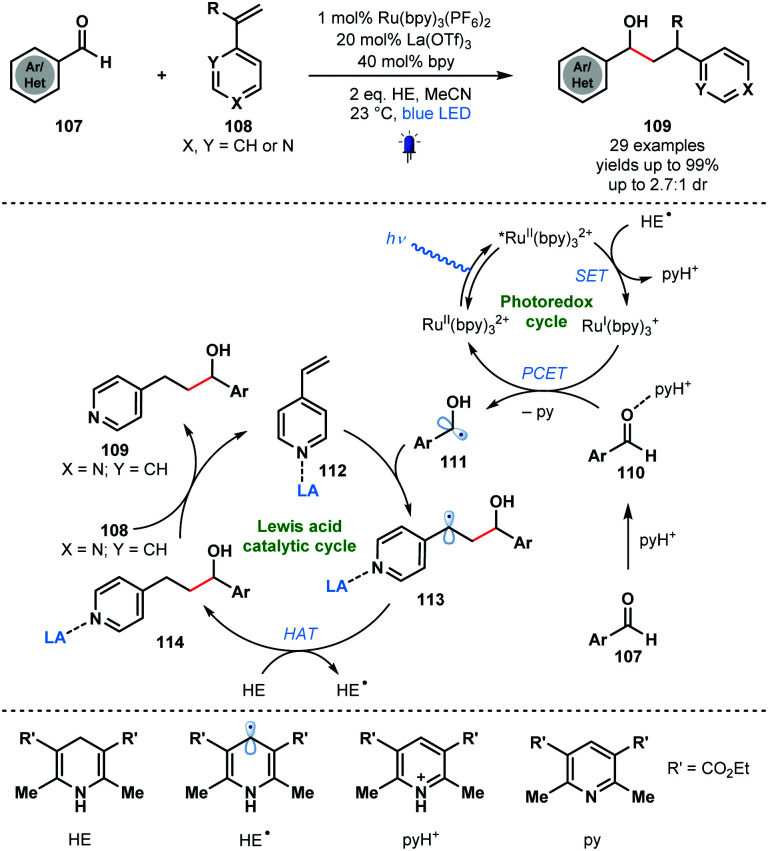
Synergistic Lewis acid/photoredox catalysis in intermolecular ketyl–olefin couplings involving aldehydes and alkenylpyridines (Ngai, 2017).

Building on the work of Knowles, Ngai and co-workers reported intermolecular photocatalytic ketyl–olefin couplings that employ a Lewis acid and a protic acid, to activate each of the coupling partners. The synergistic Lewis acid catalysis and photocatalysis allows the reductive coupling of alkenylpyridines with carbonyl and iminyl derivatives (Scheme 17).^36^ A wide range of complex substrates, including amino acid derivatives and natural products, could be converted to secondary alcohol adducts in moderate to good yield using Ru^II^(bpy)_3_(PF_6_)_2_ as the photocatalyst and La(OTf)_3_ as the Lewis acid co-catalyst. Hantzsch ester (HE) was utilised as a sacrificial electron and hydrogen atom donor. While a useful way to couple aryl aldehydes and alkenes, the method requires alkenyl pyridine partners with a Lewis basic site for activation. The proposed mechanism begins with visible light irradiation of the ground-state photocatalyst, Ru^II^(bpy)_3_^2+^ to afford Ru^II^(bpy)_3_^2+^*. The excited photocatalyst was reductively quenched by the Hantzsch ester radical (HE˙) to form Ru^I^(bpy)_3_^+^ and the corresponding pyridinium salt (pyH^+^). Aldehyde substrates **107**, activated as their hydrogen bond adducts **110** by the *in situ* generated pyH^+^, undergo a PCET process involving Ru^I^(bpy)_3_^+^ to give ketyl radicals **111**. Subsequent regioselective addition to Lewis acid-activated vinyl pyridines **112** forms stabilised radicals **113**. Hydrogen atom abstraction (HAT) from Hantzsch ester (HE) affords **114** and HE˙. Finally, the Lewis acid co-catalyst releases products **109** and activates a new molecule of substrate.

**Scheme 18:**
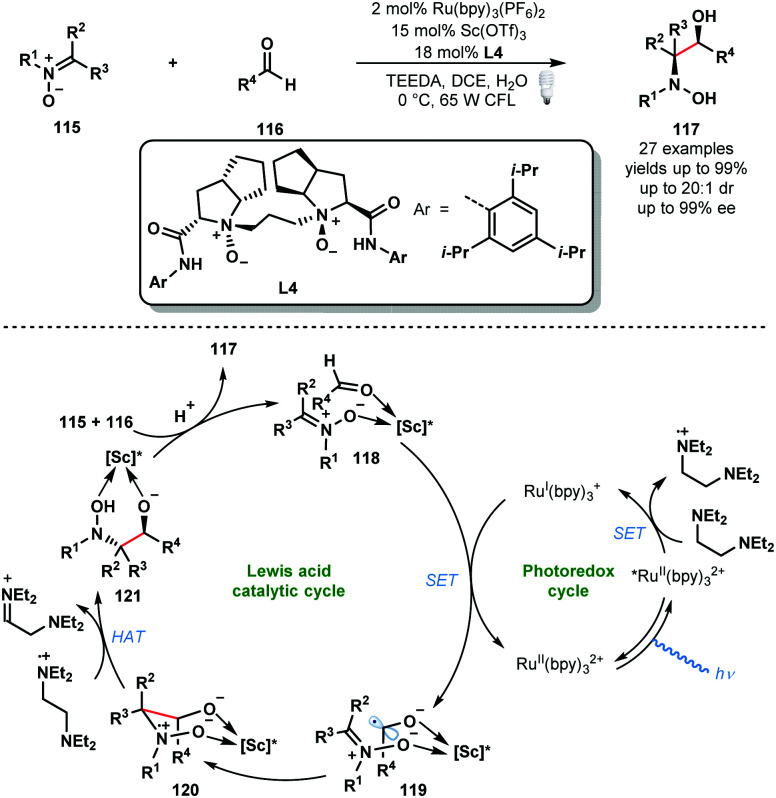
Enantioselective photocatalytic cross-coupling of nitrones and aldehydes (Huang, 2018).

Other Lewis acids can be used to activate carbonyls and aid the formation of ketyl radicals. In 2018, Huang and co-workers reported a convergent asymmetric synthesis of vicinal amino alcohols that exploits a dual Lewis acid and photocatalytic approach (Scheme 18).^37^ Analogous couplings of aldehydes/ketones and nitrones have been achieved using stoichiometric SmI_2_, however, these exploit chiral auxiliaries. A range of functionality was tolerated in the aldehyde partner and nitrone partners bearing linear, branched and cyclic alkyl motifs were successfully employed. Again, the photocatalytic cycle begins with the formation Ru^II^(bpy)_3_^2+^* by photoexcitation of Ru^II^(bpy)_3_^2+^ followed by reductive quenching of the excited state of the photocatalyst by the sacrificial reductant, *N,N,N*′*,N*′-tetraethylethylenediamine (TEEDA), producing the corresponding *N*-centred radical cation. Simultaneously, nitrone **115** and aldehyde **116** are complexed by a scandium Lewis acid derived from enantiopure ligand **L4**. Reduction of the activated aldehyde in **118** by Ru^I^(bpy)_3_^+^ delivers the complexed ketyl radical intermediate **119** and Ru^II^(bpy)_3_^2+^. This intermediate undergoes enantioselective radical addition to the bound nitrone to form the *N*-centred radical cationic intermediate **120**. Subsequent HAT from TEEDA˙^+^ affords **121** which upon protonation delivers the enantioenriched vicinal hydroxyamino alcohol product **117**. The catalytic cycle is closed by the chiral Lewis acid engaging another molecule of both **115** and **116**.

**Scheme 19:**
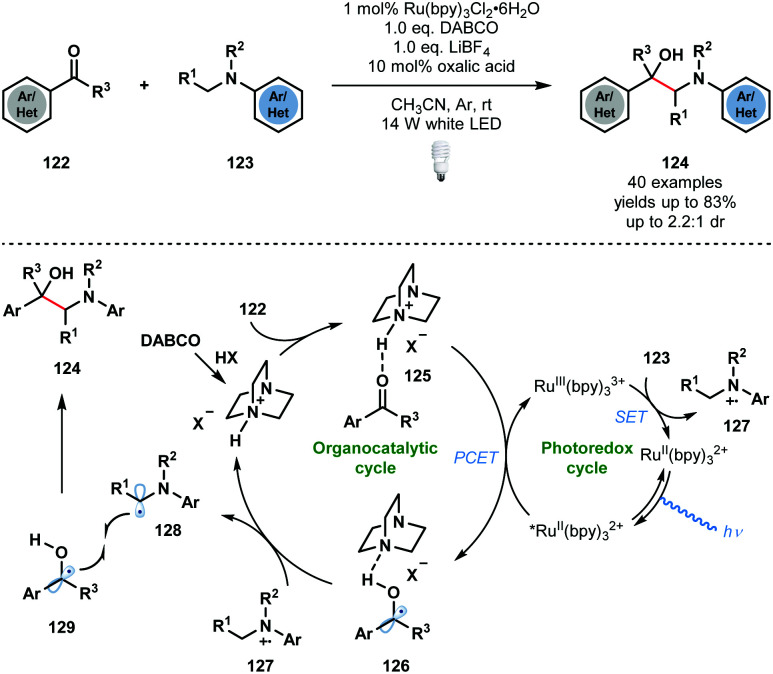
Photocatalytic cross-coupling of *N*-aryl amines with aldehydes and ketones, enabled by PCET (Wang, 2018).

In the same year, Wang and co-workers developed a complementary approach using Ru^II^(bpy)_3_^2+^ to access vicinal amino alcohols and diamines that involves the photocatalytic radical cross-coupling of aldehydes, ketones, and imines with *N*-arylamines (Scheme 19).^38^ The process exhibits good functional group tolerance, with ester, nitrile, halide, and heteroaryl substituents being compatible, however, low diastereocontrol was observed. As seen before, the carbonyl substrates must first be activated by the *in situ* generated conjugate acid of DABCO, to give complex **125**. PCET then delivers the DABCO-bound ketyl radical complex **126**. The photocatalytic cycle is closed by SET oxidation of the *N*-arylamine substrates **123** and regeneration of the ground state catalyst. Proton transfer from **127** to complex **126** yields the carbon-centred radicals **128** and **129**, as well as protonated DABCO, which undergo radical–radical coupling to afford the desired 1,2-amino alcohol products **124**. Based on ‘light on/light off’ experiments, the authors excluded a radical chain mechanism, however, such experiments are not conclusive.^28^

**Scheme 20:**
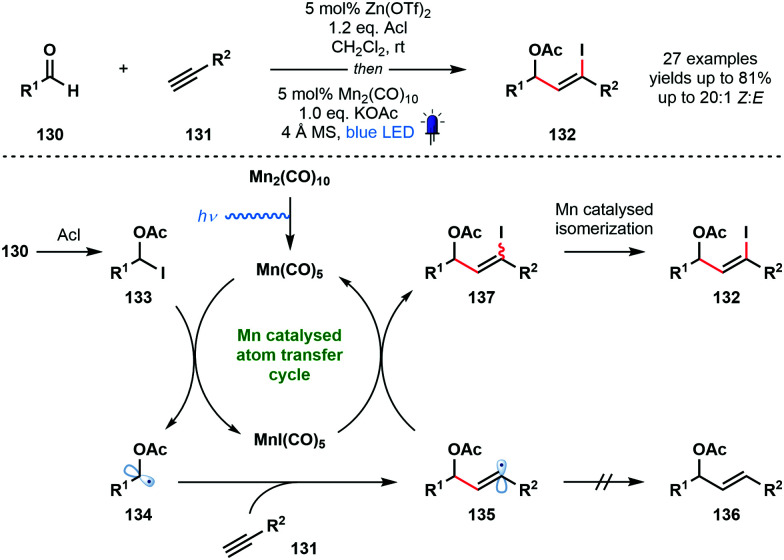
A redox neutral pathway for the generation of ketyl radical derivatives and their coupling with alkynes (Nagib, 2018).

In 2018, Nagib and co-workers reported a redox-neutral strategy that delivers α-acetoxy vinyl iodides and involves ketyl radical derivatives (Scheme 20).^39^ The method is simple, uses commercial coupling partners, and yields synthetically useful vinyl iodides with high *Z*-selectivity. Interestingly, even formaldehyde can be used to generate the corresponding α-acetoxy vinyl iodide after coupling with TMS-acetylene. While steric factors prevented this method being extended to simple ketones, trifluoroacetone was used successfully. Furthermore, electron-rich alkenes, in place of alkynes, also underwent successful coupling. Most photocatalysed methods that deliver ketyl radicals use a sacrificial terminal reductant, such as an amine. In this redox-neutral approach, the carbonyl reduction step is replaced by a halogen atom abstraction step that forms ketyl radical derivatives. To close the catalytic cycle, the halogen atom is returned and product is formed. Acetyl iodide, in the presence of a zinc Lewis acid catalyst, activates aldehydes **130** as adducts **133**, and visible light-mediated homolytic cleavage of Mn_2_(CO)_10_ generates a 17-electron complex, Mn(CO)_5_. This species readily accepts I˙ from the weak C–I bond of **133**. The resulting ketyl radical derivatives **134** add to the terminal alkyne partners **131** to form vinyl radical intermediates **135**. These intermediates are quenched by MnI(CO)_5_ to regenerate the active Mn(CO)_5_. Importantly, this atom transfer radical addition (ATRA) mechanism precludes the formation of reduced product **136**. Finally, a Mn-catalysed isomerization *via* vinyl radical intermediates ensures that high *Z*-selectivity is seen in the formation of products **132**. An alternative, radical chain mechanism was deemed unlikely based on chain initiation and cross-over experiments.

**Scheme 21:**
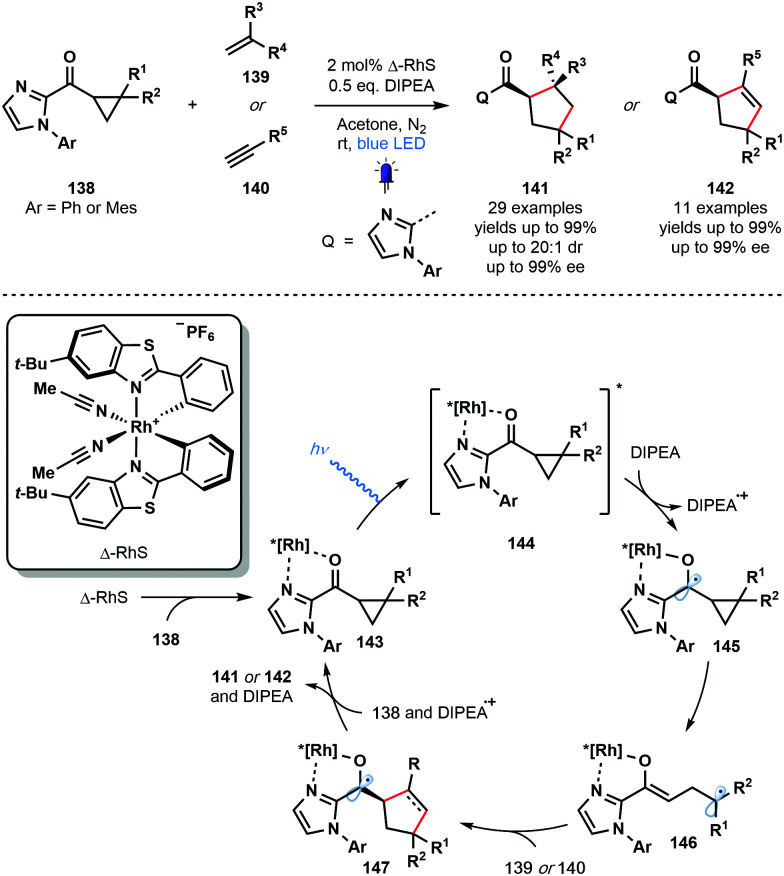
Enantioselective, Rh-catalysed, visible light-mediated [3+2] cycloaddition of cyclopropyl ketones and alkenes or alkynes (Meggers, 2018).

Also in 2018, Meggers and co-workers employed a chiral-at-metal photocatalyst (Δ-RhS) in a photocatalytic enantioselective [3+2] photocycloaddition involving cyclopropyl ketones and alkenes or alkynes (Scheme 21).^40^ Enantioenriched cyclopentane- and cyclopentene-derivatives were synthesized in excellent yield with excellent levels of enantio- and diastereocontrol. Various unsaturated partners were successful ranging from simple acrylates to highly functionalized drug derivatives. Two-point coordination of the cyclopropyl ketone substrates **138** affords complexes **143** which are excited by visible light to give **144**. This strongly oxidising species undergoes SET reduction by DIPEA to give the ketyl radical intermediates **145** which subsequently fragment to form Rh-bound enolate radicals **146**. Intermolecular radical addition to the alkene or alkyne partners, **139** or **140**, respectively, is followed by an enantiodetermining radical cyclisation event – controlled by the helical chirality of the Rh complex – that yields highly-reducing ketyl radical complexes **147**. Back electron donation to DIPEA˙^+^ and ligand exchange regenerates complexes **143** and liberates products **141** or **142**. Alternatively, quantum yield measurements supported a radical chain process in which ketyl radicals **147** can directly reduce excited complexes **144**.

**Scheme 22:**
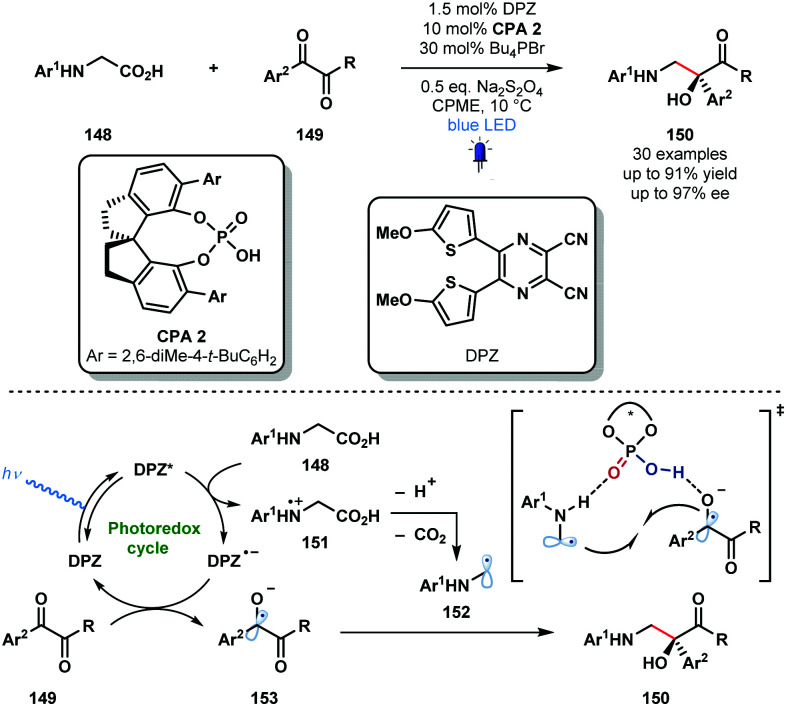
Enantioselective, photocatalytic radical-coupling of activated ketones and *N*-aryl glycines (Jiang, 2018).

Organic photocatalysts can also be used to generate ketyl radicals from activated ketones. In 2018, the Jiang group reported a redox-neutral approach to valuable, enantioenriched 1,2-amino alcohols **150** from *N*-aryl glycines **148** and vicinal diketones **149** using photocatalyst DPZ in conjunction with enantiopure **CPA2** which serves as a dual H-bond donor and acceptor catalyst (Scheme 22).^41^ This radical–radical coupling shows good functional group tolerance and can be extended to isatin partners. The high yields and high enantiocontrol observed renders this a practical enantioselective approach to vicinal aminoalcohols.

Glycine derivatives **148** are readily oxidised by the excited dicyanopyrazine-derived photocatalyst DPZ to afford α-amino radicals **152** after deprotonation and decarboxylation. Activated ketones **149** are then reduced to close the photocatalytic cycle yielding ketyl radical intermediates **153**. Double H-bonding interactions are thought to facilitate capture of **152** and **153** by **CPA2** and enantioselective radical–radical coupling gives the desired products **150** in good yield and with excellent enantiocontrol.

**Scheme 23:**
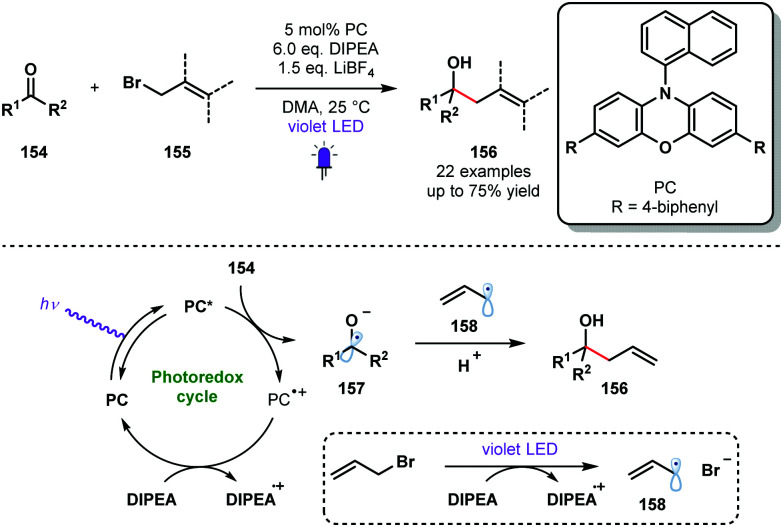
Violet light-mediated allylation and benzylation of aldehydes and ketones (König, 2018).

Violet light has also been used to generate ketyl radicals from aldehydes and ketones. In 2018, König reported a photocatalytic synthesis of homoallylic and homobenzylic alcohols from readily available allyl or benzyl bromides and aromatic aldehydes or ketones that is reminiscent of a Barbier-type coupling (Scheme 23).^42^ Yields were moderate in many cases, possibly due to competing pinacol formation; products of homocoupling were observed in some cases. However, this process does allow the use of stoichiometric metal reagents, typically associated with Barbier-type processes, to be avoided. The reductive radical–radical cross-coupling reaction uses phenoxazine-derived organic photocatalyst PC and DIPEA as a sacrificial electron donor and proton source. Mechanistic investigations suggested that the reduction potential of carbonyl partners **154** were significantly reduced by simultaneous activation by DIPEA and LiBF_4_. The excited photocatalyst PC* reduces carbonyl species **154** to deliver the ketyl radical species **157**. DIPEA closes the photoredox cycle and is thought to act as a SET reductant during the formation of stabilised radicals **158** from, for example, allyl bromides. Radical–radical coupling between **157** and **158** delivers the alcohol products **156** in good yield.

**Scheme 24:**
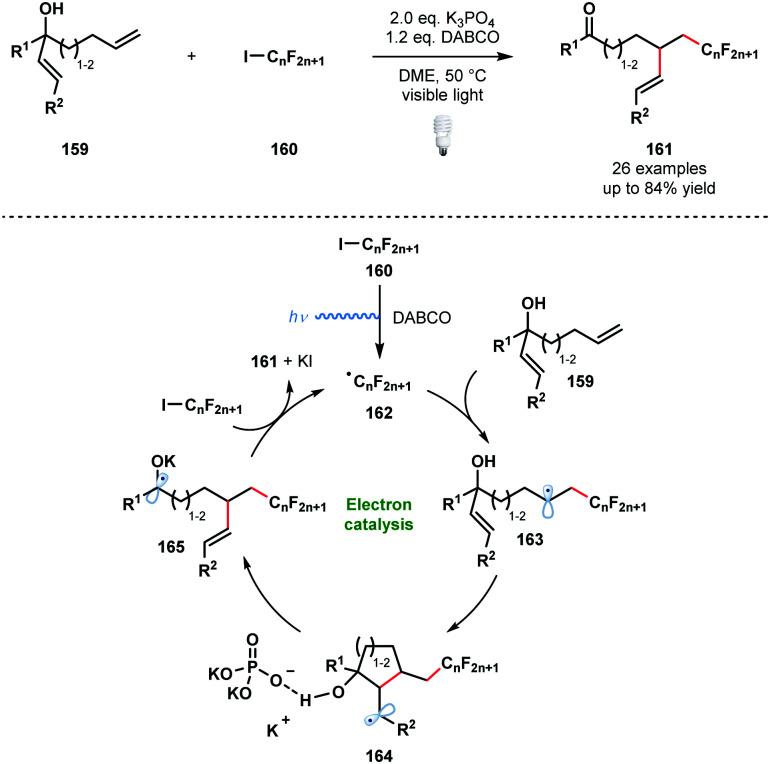
Visible light-initiated 1,2-functionalisation of alkenes by radical perfluoroalkylation/alkenyl migration (Studer, 2018).

In 2014, Studer and Curran introduced the term ‘electron catalysis’ to describe SET chain and relay processes.^43^ Four years later, Studer and co-workers exploited such a process in an efficient method for the α-perfluoroalkylation/β-alkenylation of unactivated alkenes that is driven by the formation of ketyl radical anions (Scheme 24).^44^

Visible light irradiation of the halogen-bond complex between DABCO and **160** affords the perfluoroalkyl radicals **162** and initiates the process. Radicals **162** add to the more accessible terminal alkene to give **163**. Radical 5-*exo*-trig or 6-*exo*-trig cyclisation leads to intermediates **164** bound to an orthophosphate anion by hydrogen bonding. This interaction is thought to be key in activating the β-C–C bond towards homolytic cleavage. Concurrent deprotonation and β-C–C bond cleavage affords ketyl radical **165** which reduces **160** to sustain the chain process and yield the 1,2-functionalised alkenes **161**. An analogous α-perfluoroalkyl-β-alkynylation of alkenes was reported earlier by the same team.^45^

**Scheme 25:**
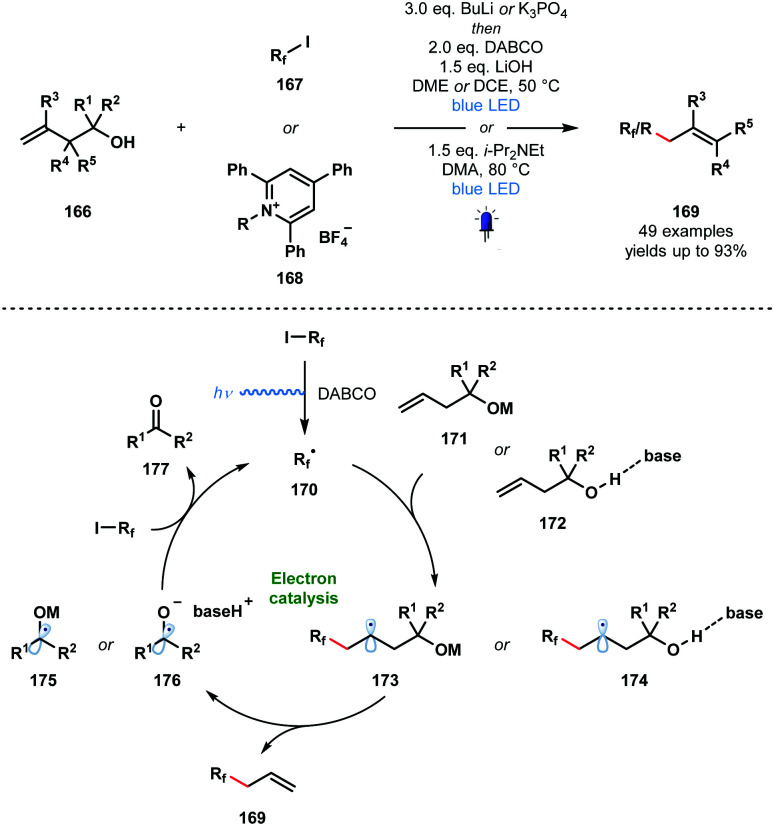
Visible light-initiated radical allylation using homoallylic alcohols and base (Studer, 2020).

In 2020, Studer and co-workers applied their approach to the visible light-initiated allylation of radicals using homoallylic alcohols (Scheme 25).^46^ In this variant, perfluoroalkyl radicals and simple alkyl radicals – formed from pyridinium salts **168** – were generated and engaged in radical additions to homoallylic alcohols **166** to yield allylated products **169** after base-promoted homolytic cleavage of the C_α_–C_β_ bond. This SET chain process is sustained by the reduction of **167** or **168** by the resultant ketyl radicals **175** or **176**.

**Scheme 26:**
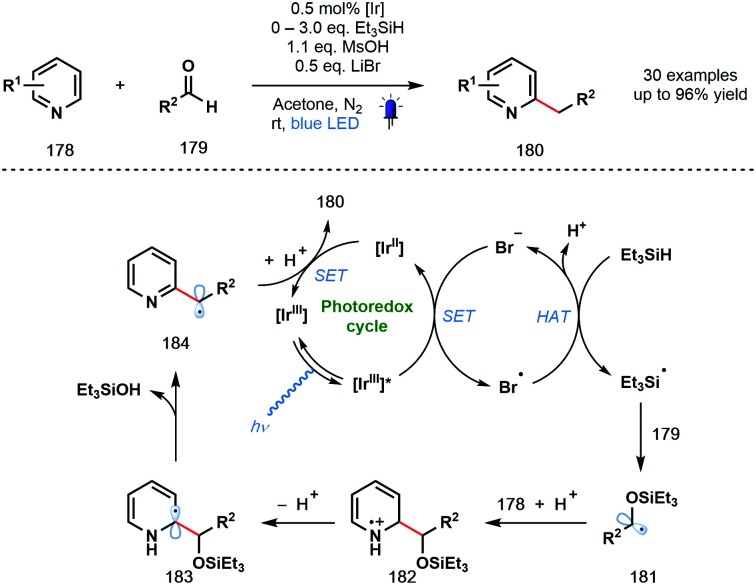
Photocatalytic Minisci-type reactions using ketyl-radical derivatives generated from aldehydes (Huang, 2020).

In 2020, the Huang group described a photocatalytic Minisci-type reaction that exploits simple aldehydes as alkylating agents (Scheme 26).^47^ In particular, under the mild reaction conditions, products of the benzylation of heteroarenes can be obtained; an elusive process in Minisci chemistry. The reaction tolerates a wide range of functionalities, however, the two simple pyridine substrates tested – 2-Ph-pyridine and 2-Bn-pyridine – gave low yields of the corresponding benzylated products. The excited photocatalyst [Ir^III^]* is thought to be reductively quenched by bromide to afford Br˙ which abstracts a hydrogen atom from Et_3_SiH to form silyl-centred radical Et_3_Si˙. Coupling of this silyl radical to aldehydes **179** afforded ketyl radical derivatives **181** that undergo subsequent radical addition to protonated pyridine derivatives **178** to yield radical cations **182**. Deprotonation and loss of Et_3_SiOH delivers carbon-centred radicals **184**. Finally, SET from [Ir^II^] followed by protonation liberates the alkylated products **180**.

## 5. Electrochemically generated ketyl radicals

**Scheme 27:**
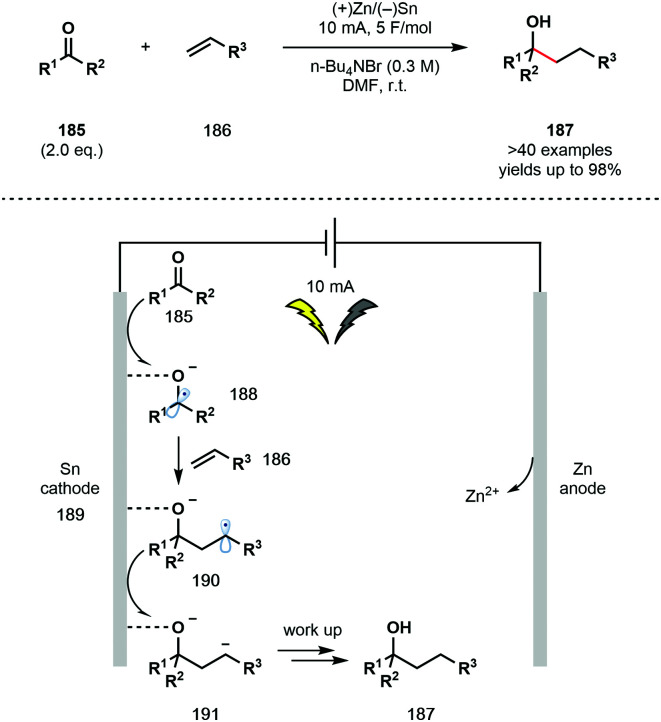
Electrochemical ketyl–olefin coupling using unactivated ketones and unactivated alkenes (Baran, 2020).

Baran *et al.* have recently developed a user-friendly, electrochemical, intermolecular ketyl–olefin coupling using an undivided cell setup (Scheme 27).^48^ Previous electrochemical methods reported by Shono and co-workers mainly focused on intramolecular processes.^48^ Furthermore, past reports were limited by the challenges of working with a divided cell setup under an inert atmosphere and the requirement for a significant excess of the ketone partner. The new protocol allows for the use of simple alkenes **186** and unactivated ketones **185**, and thus complements known protocols using SmI_2_, and other single-electron reducing agents, that require activated alkene partners in an intermolecular setting. Under the optimised reaction conditions, an inexpensive sacrificial anode (Zn) was used in combination with only two equivalents of the ketone partner. Conveniently, the reaction uses an undivided cell, can be run under air, no precautions to exlude moisture are necessary, and the process has been shown to tolerate a wide range of functional groups. While ketones bearing α-heteroatom substituents proved to be ineffective partners, their fragmentation provided useful support for the intermediacy of a ketyl radical. Importantly, the strong adsorption of **188** to the Sn cathode changes its electronic properties and reactivity and thus facilitates its addition to unactivated alkene coupling partners **186**.

## 6. Ketyl radicals in natural product synthesis

**Scheme 28:**
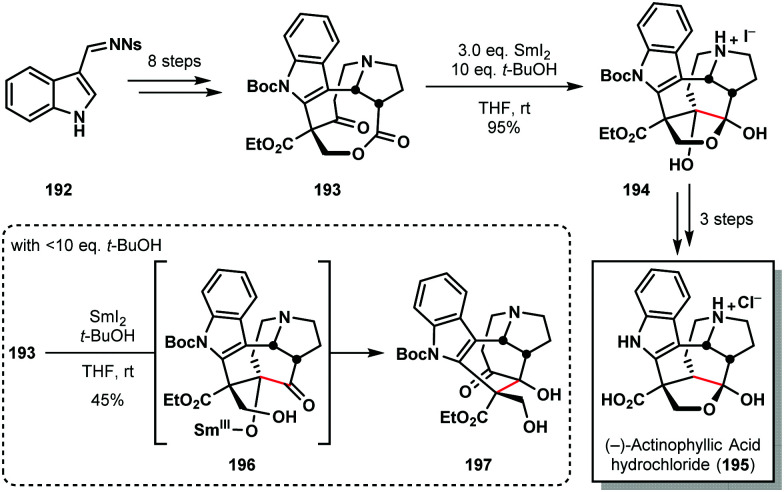
A ketyl radical in the pivotal ketone–ester pinacol coupling en route to (−)-actinophyllic acid (Kwon, 2016).

The chemistry of ketyl radicals, accessed in various ways, continues to play a key role in total synthesis. The reagent's versatility means that SmI_2_ is often the reagent of choice in many studies.^49^ For example, Kwon's 2016 synthesis of (−)-actinophyllic acid (**195**) compares the suitability of various SET reductants for the pivotal step in the synthesis; a ketone–ester pinacol coupling (Scheme 28).^50^ Several reagents, including those based on Ti(iii), Li, and Na, failed to convert **193** to the desired product **194**. However, SmI_2_, in conjunction with 10 eq. of *t*-BuOH, delivered the caged scaffold of **195** in nearly quantitative yield. Interestingly, the amount of proton source proved crucial; the use of >10 eq. of *t*-BuOH reduced the reaction rate and <0 eq. of *t*-BuOH favoured the formation of rearranged product **197**, presumably *via* intermediate **196** which then underwent a retro-aldol/aldol cascade.

**Scheme 29:**
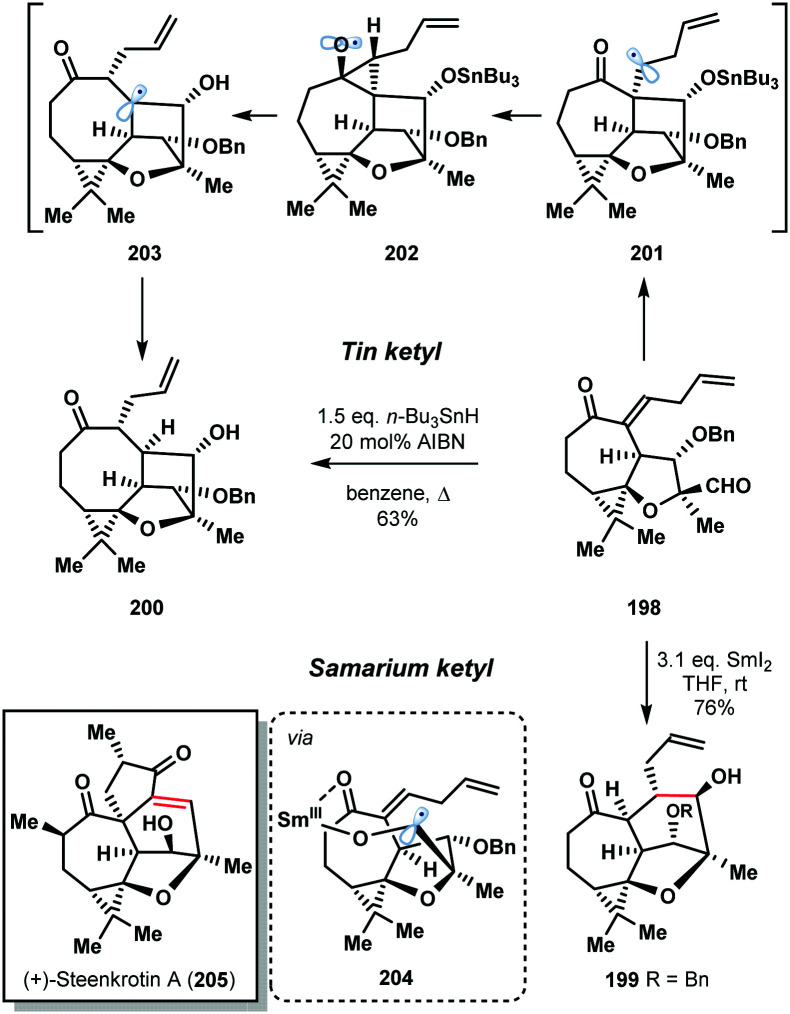
Contrasting the reactivity of tin and samarium ketyl radicals in the total synthesis of (+)-steenkrotin A (Ding, 2016).

The 2016 enantioselective total synthesis of (+)-steenkrotin (**205**) A by Ding *et al.* provides a striking illustration of the differences in behaviour of tin and samarium ketyl radicals (Scheme 29).^51^ The synthetic plan relied on a ketyl–olefin cyclisation of aldehyde **198** to build the tetracyclic core of the natural product **199**. Rather than delivering **199**, treatment of **198** with *n*-Bu_3_SnH in refluxing benzene gave undesired, rearrangement product **200** in 63% yield. Formation of **200** involves conversion of the aldehyde to the tin ketyl radical followed by intramolecular addition to the enone in an undesired 5-*exo*-trig fashion to form radical **201**. A Beckwith–Dowd ring expansion, *via* cyclopropane **202**, gives radical **203** which is quenched by *n*-Bu_3_SnH. The desired 6-*endo*-trig cyclization was achieved in a highly stereoselective manner by treatment of aldehyde **198** with SmI_2_ at room temperature. The oxophilic Sm^III^ ion of the ketyl radical coordinates to the enone moiety, controlling the regioselectivity of the cyclization by lowering the energy of the π* orbital of the olefin (**204**).

In the context of total synthesis, the most impressive applications of SmI_2_ for ketyl radical generation result in cascade processes that assemble complex architectures with exquisite control.^49^

**Scheme 30:**
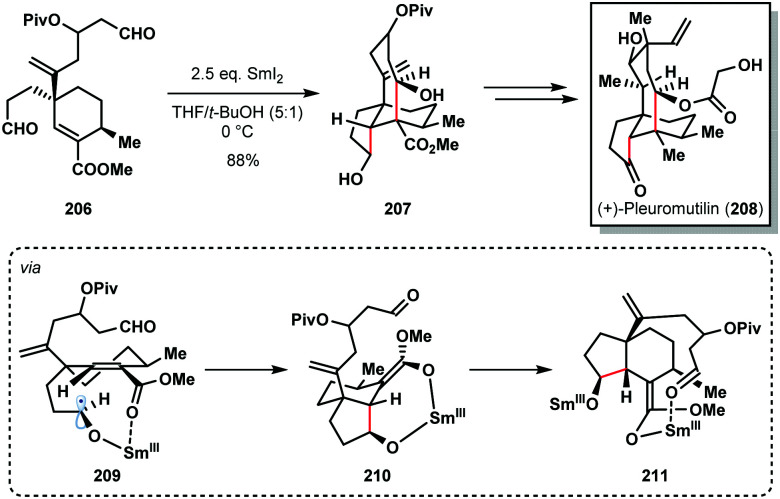
SmI_2_-mediated ketyl radical formation in a cyclization cascade en route to (+)-pleuromutilin (Procter, 2013).

For example, Procter's total synthesis of (+)-pleuromutilin (**208**) features a SmI_2_-mediated radical cascade cyclisation that forges the five- and eight-membered rings of the target and sets four contiguous stereocentres with high diastereocontrol (Scheme 30).^52^ The selective conversion of dialdehyde **206** to tricycle **207**, using SmI_2_ in the presence of *t*-BuOH, proceeded in 88% yield on gram scale. This cascade reaction again highlights the high control often seen in SmI_2_-mediated ketyl radical reactions that arises from coordination of samarium to Lewis basic sites in the substrate; chemoselective formation of ketyl radical **209**, a chelation-controlled *anti*-5-*exo*-trig cyclization (delivering **210**), and a diastereoselective aldol cyclization of Sm(iii)-enolate **211** furnishes **207**.

**Scheme 31:**
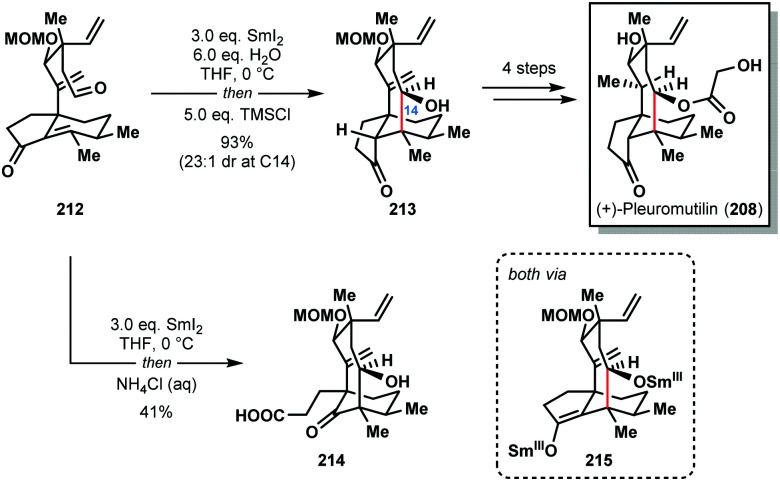
A SmI_2_-mediated ketyl–olefin cyclization in the total synthesis of (+)-pleuromutilin (Reisman, 2018).

In 2018, Reisman and co-workers also achieved the synthesis of (+)-pleuromutilin (**208**) by using the SmI_2_–H_2_O reagent system to construct the eight-membered ring of the natural product with high diastereocontrol (Scheme 31).^53^ Interestingly, treatment of aldehyde **212** with SmI_2_, followed by work-up under air, gave undesired carboxylic acid **214** in 41% yield; exposure of Sm(iii)-enolate intermediate **215** to oxygen results in the formation of an α-peroxyketone that undergoes oxidative ring scission. However, under rigorously anaerobic conditions, trapping of enolate **215** with TMSCl, followed by aqueous work up, provided the desired tricycle **213** in 93% yield with near perfect diastereocontrol. In the absence of water, the ketyl–olefin cyclization resulted in a 1 : 1 mixture of diastereomers at C14.

**Scheme 32:**
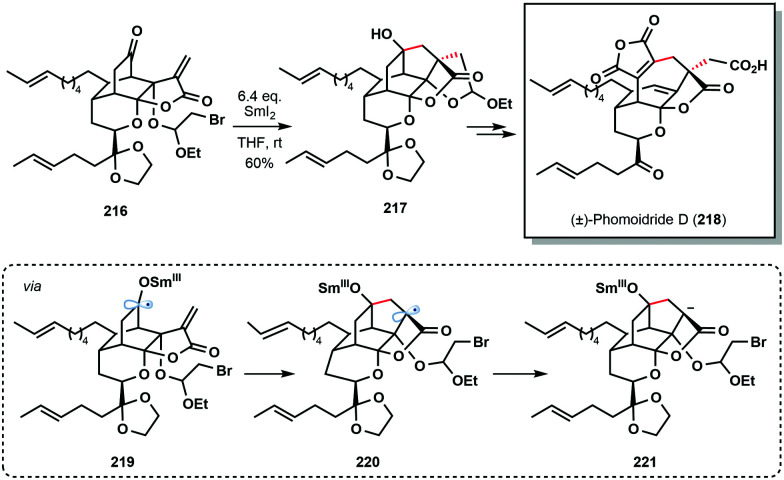
SmI_2_-mediated ketyl radical formation in a cyclization cascade approach to (±)-phomoidride D (Wood, 2018).

Another prime example of a cascade reaction employing a ketyl radical generated by SmI_2_ comes from the 2018 total synthesis of (±)-phomoidride D (**218**) by Wood and co-workers (Scheme 32).^54^ Treatment of ketone **216** with SmI_2_ triggered a 5-*endo*-trig/5-*exo*-tet cyclization sequence; 5-*endo*-trig cyclization of ketyl radical **219**, followed by reduction, and an unusual intramolecular alkylation of an organosamarium (**221**), delivered advanced intermediate **217**.

**Scheme 33:**
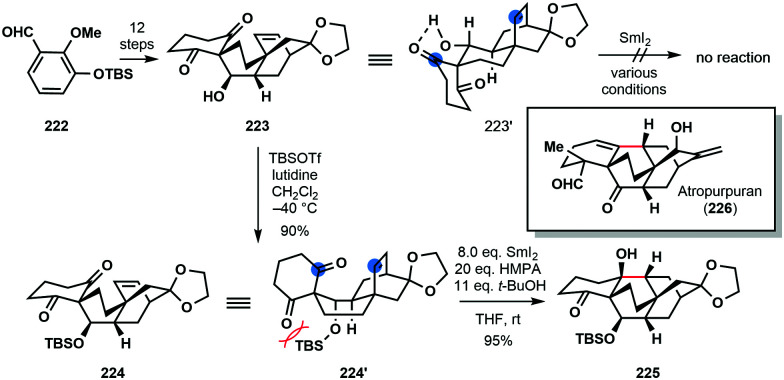
Control of conformation in a SmI_2_-mediated ketyl–olefin cyclization en route to (±)-atropurpuran (Qin, 2016).

In the 2016 total synthesis of (±)-atropurpuran (**226**) by Qin and co-workers, an understanding of conformation proved key in the development of a SmI_2_-mediated ketyl–olefin cyclization (Scheme 33).^55^ Upon exposure to SmI_2_ under a variety of conditions, the proposed radical cyclization of secondary alcohol **223** proved unsuccessful and only starting material was returned. It was postulated that **223** adopts chair conformation **223′**, featuring an intramolecular hydrogen bond between the hydroxyl and a ketone carbonyl; thus, the ketyl radical, formed upon ketone reduction, and the alkene partner are not sufficiently close for coupling. Protection of **223** as a bulky TBS ether breaks up the hydrogen bond interaction and switches the conformation of **224** to that of a boat (**224′**). Treatment of TBS-protected alcohol **224** with SmI_2_ in the presence of HMPA resulted in smooth cyclization to deliver the core of the natural product **225** in 95% yield.

**Scheme 34:**
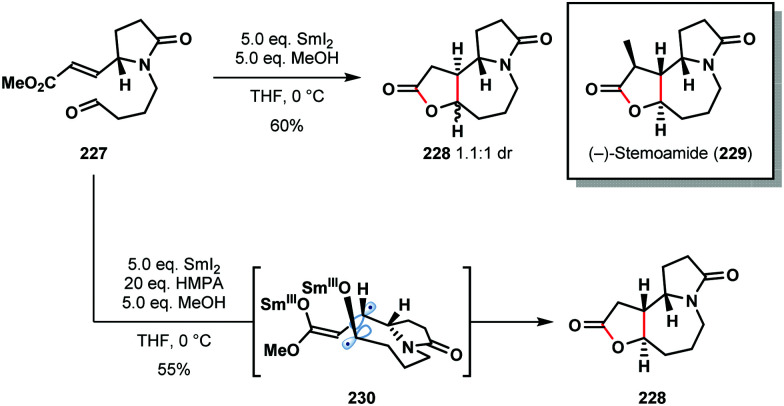
The effect of HMPA on a SmI_2_-mediated ‘ketyl–olefin’ cyclization in the total synthesis of (−)-stemoamide (Honda, 2011).

It is well-known that additives, such as HMPA and proton sources, play a crucial role in SmI_2_-mediated reactions. For example, HMPA can not only promote couplings but can also alter the stereoselectivity of C–C bond formation. Honda's 2011 total synthesis of (−)-stemoamide (**229**) provides an illustration of the dramatic impact HMPA can have on SmI_2_-mediated ketyl–olefin couplings (Scheme 34).^56^ In the absence of HMPA, aldehyde **227** underwent cyclization to give **228** as a 1.1 : 1 diastereoisomeric mixture in 60% yield. By employing HMPA in conjunction with SmI_2_, **228** was obtained as a single diastereomer in 55% yield. Interestingly, the selective coupling of diradical intermediate **230** was proposed; HMPA is known to significantly increase the reduction potential of SmI_2_ and may allow access to diradical **230**. It is also likely that HMPA breaks up chelated transition states that lead to the alternative diastereoisomer. Finally, the coupling of *Z*-**227** gave a similar reaction outcome; this supports an ‘alkene first’ mechanism in which the alkene geometry is lost prior to coupling and thus has little effect on the product distribution.

## 7. Conclusions

Ketyl radicals are highly versatile reactive intermediates, well-known for their synthetic utility in C–C bond forming reactions and their ability to forge complex molecular architectures. Ketyl radicals are still commonly generated using stoichiometric amounts of SET reducing agents – such as K, Zn, Ti and, most importantly, SmI_2_ – and these processes continue to underpin the pivotal steps in many high profile total syntheses. However, the advent of photoredox catalysis and the growth of transition–metal catalyzed radical processes, in particular, has led to a renaissance in the chemistry of ketyl radicals. For example, striking advances in the field exploit visible light-mediated photocatalytic SET, PCET, HAT, and halogen atom transfer processes that deliver ketyl radicals for C–C bond formation. Finally, recent innovative reports have described how the enantioselectivity of reactions involving ketyl radicals can be controlled. We look forward to further advances in the generation and selective harnessing of ketyl radicals, particular in the context of sustainable catalysis, and increased application of the versatile reactive intermediates to synthetic challenges in both academic and industrial settings.

## Conflicts of interest

There are no conflicts to declare.
